# How the Sino–U.S. Trade War Rewired Global Soybean Price Linkages: Time-Varying Spillovers and Frequency-Domain Evidence

**DOI:** 10.3390/foods15101678

**Published:** 2026-05-11

**Authors:** Qi Zhang, Yi Hu, Yao Yue

**Affiliations:** 1 Business School, Beijing Technology and Business University, Beijing 100048, China; zhangqi@btbu.edu.cn; 2 School of Economics and Management, University of Chinese Academy of Sciences, Beijing 100190, China; 3 MOE Social Science Laboratory of Digital Economic Forecasts and Policy Simulation, University of Chinese Academy of Sciences, Beijing 100190, China; 4 Agricultural Bank of China, Beijing 100005, China; yueyao19@mails.ucas.ac.cn

**Keywords:** soybean market, risk spillovers, Sino–U.S. trade war, supply-chain reconfiguration

## Abstract

Soybeans are a strategic commodity in global agricultural trade, and disruptions to their pricing system have direct implications for food security and trade patterns. This paper examines how major external shocks, particularly the Sino–U.S. trade wars, reshaped the dynamic connectedness and risk transmission structure of the global soybean price system. Using daily data from 2015–2025 for five key benchmarks, Chicago Board of Trade (CBOT) soybean futures, Dalian Commodity Exchange (DCE) No. 1 soybean futures, and cost-and-freight (CNF) prices for U.S. Gulf, Brazil, and Argentina shipments to China, we apply the time-varying parameter vector autoregression Diebold–Yilmaz connectedness model (TVP-VAR-DY) and the time-varying parameter vector autoregression Baruník–Křehlík frequency connectedness model (TVP-VAR-BK) models to quantify time-varying spillovers across short-, medium-, and long-run horizons. The results indicate that the global soybean market is highly integrated, while systemic risk transmission is predominantly short-run and declines sharply at longer horizons. CBOT futures remain the principal source of spillovers, especially in the short term, yet their net influence weakens noticeably after the 2018 trade-friction episode and declines further following the 2025 episode, particularly with respect to South American CNF benchmarks. Frequency-specific evidence suggests that trade-policy escalations are increasingly priced as structural shocks, strengthening medium- and long-horizon connectedness, while DCE’s outward spillovers rise markedly around 2025, consistent with the emergence of a more regionalized pricing architecture centered on Chinese demand. Within South America, Brazil leads short-run price formation, whereas longer-horizon dynamics are more exposed to Argentine policy risk spillovers. These findings provide new evidence on supply-chain reconfiguration and benchmark rebalancing in global soybean pricing and offer policy implications for strengthening China’s pricing capacity and enhancing multi-horizon supply-chain risk management.

## 1. Introduction

Since the early 2000s, the increasing integration of global agricultural commodity markets has played a central role in food security and international trade. Among major agricultural commodities, soybeans occupy a particularly strategic position, as they are essential not only for international trade but also for edible oil processing, livestock feed production, and the stability of broader agri-food supply chains. Globally, soybean production exceeds 350 million tons annually, with Brazil and the United States accounting for the majority of exports, while China alone represents the dominant share of global import demand. Soybeans are also widely processed into soybean oil and soybean meal, which are critical inputs in food industries and animal production. The global soybean trade network links the Americas’ production belt with East Asia’s consumption hub, making soybean price formation and cross-market risk transmission a central issue in the functioning of the agri-food system. This market structure, characterized by concentrated export origins and strong import dependence, makes soybean trade particularly sensitive to policy shocks and supply-chain disruptions.

For decades, global soybean pricing has largely revolved around CBOT soybean futures, complemented by origin-specific basis adjustments reflecting transportation costs, quality differences, and local supply–demand conditions. This system historically exhibited a relatively stable and centralized structure, with CBOT acting as the dominant benchmark and primary transmitter of price information across markets. However, the Sino–U.S. trade tensions that began in 2018 introduced a major policy shock to this system. In response to tariff escalation, China—the world’s largest soybean importer—significantly restructured its sourcing strategy, shifting imports away from the United States toward Brazil and other South American suppliers. By 2024, Brazilian soybeans accounted for more than 70% of China’s total soybean imports, while the U.S. share fell to below 20%. This large-scale reallocation of trade flows not only altered bilateral trade patterns but also raised the possibility that the underlying structure of global soybean price formation and risk transmission has been fundamentally reshaped.

Importantly, this transformation cannot be interpreted as a one-off adjustment. Rather, it reflects an evolving process shaped by repeated trade-policy shocks, origin-specific basis movements, and ongoing supply-chain adaptation. Weather disruptions, logistics constraints, and port conditions can periodically amplify landed-cost volatility and reinforce incentives to diversify sourcing beyond Brazil toward other South American origins, including Argentina. At the same time, trade-policy uncertainty has become increasingly persistent. In particular, the renewed escalation of Sino–U.S. trade tensions in April 2025 differed materially from the 2018 episode in both speed and intensity, further increasing uncertainty over soybean sourcing, pricing, and cross-market risk transmission. In this context, the key issue is not merely whether prices reacted to tariff shocks, but whether successive trade-war episodes have altered the architecture of cross-market connectedness and risk transmission.

Existing studies have examined the effects of the Sino–U.S. trade war on soybean markets, but the literature remains fragmented. On the one hand, studies on trade flows document significant trade diversion and import substitution. On the other hand, research on futures markets focuses on price discovery, volatility spillovers, and market reactions. However, these strands of literature are rarely integrated into a unified analytical framework, and most studies treat trade frictions as a single, static shock. As a result, there is limited evidence on whether repeated trade-war episodes have reshaped pricing influence across the broader soybean pricing system, particularly through the interaction between physical trade reallocation and financial price transmission.

Accordingly, the unresolved issue is not simply whether soybean prices changed during trade conflicts, but whether these shocks led to a persistent reorganization of global soybean price connectedness. Addressing this issue requires an empirical framework that can capture both the time-varying evolution of spillovers and their heterogeneity across different horizons.

Building on this gap, the present study addresses three interrelated research questions. First, did the Sino–U.S. trade war reshape the structure of risk transmission within the global soybean pricing system? Second, did the renewed escalation in 2025 differ from the 2018 episode in terms of pricing influence, market connectedness, and spillover patterns? Third, are the observed spillovers primarily short-lived responses to policy shocks, or do they reflect more persistent structural adjustments associated with supply-chain reconfiguration and changing import dependence?

To answer these questions, we adopt a unified time-varying parameter vector autoregression (TVP-VAR) framework and analyze daily prices from five key markets: CBOT soybean futures as the global benchmark, DCE soybean futures as a proxy for China’s domestic market, and China-bound landed prices (CNF) representing import costs from the United States, Brazil, and Argentina. Within this framework, we employ two complementary connectedness measures. The TVP-VAR-DY model is used to quantify the time-varying magnitude and direction of spillovers across markets, allowing us to identify net transmitters and receivers of risk over time. Building on this, the TVP-VAR-BK model further decomposes these spillovers across different frequency bands, enabling us to distinguish between short-run responses to policy shocks and longer-run structural adjustments associated with supply-chain reconfiguration. These two approaches are therefore complementary rather than redundant, jointly providing a more comprehensive understanding of the evolution and persistence of risk transmission.

This study contributes to the literature in three respects. First, it moves beyond the dominant focus on futures-to-futures transmission by jointly modeling benchmark futures, domestic futures, and origin-specific landed prices, thereby linking trade reallocation to changes in pricing influence within the global soybean system. Second, it employs a unified TVP-VAR framework that integrates time-domain and frequency-domain connectedness, allowing us to capture both the dynamic evolution and persistence of spillovers. Third, it provides new empirical evidence that successive trade-war episodes are associated with a weakening of CBOT-centered pricing dominance and the emergence of a more regionally differentiated and multi-centered connectedness structure.

The remainder of the paper is organized as follows. Section 2 reviews the related literature. Section 3 describes the data and outlines the TVP-VAR-DY and TVP-VAR-BK methodologies. Section 4 presents the empirical results and discussion. Section 5 reports robustness tests. Section 6 concludes and discusses policy implications.

## 2. Literature Review

### 2.1. Trade War and Commodity Markets

The Sino–U.S. trade war has generated extensive research on tariff escalation, trade diversion, and commodity-market adjustment. Among agricultural commodities, soybeans occupy a central position due to their strong global demand, highly concentrated export supply (primarily the United States and Brazil), and China’s dominant role as the largest importer. These characteristics make the soybean market particularly suitable for examining how trade-policy shocks affect not only trade flows but also the broader process of global price formation.

A large body of literature shows that tariff escalation and trade policy uncertainty have significant price and welfare effects, suggesting that trade-policy shocks should be understood as price-relevant disturbances rather than purely institutional changes [1,2,3]. Agricultural markets are especially sensitive to such shocks due to biological production cycles, seasonal constraints, and limited short-run supply flexibility [4,5].

Empirical evidence further shows that trade frictions led not only to reductions in bilateral trade but more importantly, to substantial trade reallocation. In the soybean market, China’s retaliatory tariffs sharply reduced imports from the United States and redirected demand toward alternative suppliers, particularly Brazil, thereby generating persistent origin-specific price effects and broader market adjustments [6,7]. Beyond physical trade, recent studies document that trade tensions also affected futures-market dynamics, including price discovery and volatility transmission, indicating that trade-policy shocks propagate through both real and financial channels [8,9]. From a broader perspective, these developments are associated with a reconfiguration of the global soybean trade network, which may alter cross-market linkages and the transmission of shocks across regions [10].

Despite these advances, the literature remains fragmented in several respects. Existing studies tend to examine either trade flows or price dynamics but rarely integrate the two within a unified framework. Moreover, it remains unclear whether the observed changes reflect temporary adjustments during trade conflicts or a more persistent reorganization of global market structure. In particular, there is limited evidence on how trade reallocation interacts with financial price transmission to reshape global soybean price connectedness.

### 2.2. Spillovers

The spillover literature provides the main analytical framework for understanding how shocks propagate across markets. Early studies relied on correlation analysis, Granger causality, and multivariate generalized autoregressive conditional heteroscedasticity (GARCH) models to capture interdependence and volatility transmission. While these approaches offer useful insights, they have limitations in identifying directional spillovers and system-wide risk transmission, particularly under structural change.

To address these limitations, Diebold and Yilmaz [11,12,13] developed the connectedness framework based on generalized forecast error variance decompositions from vector autoregressions. This approach quantifies total, directional, and net spillovers, providing an intuitive and economically interpretable measure of risk transmission across markets. However, the standard Diebold–Yilmaz connectedness (DY) framework typically assumes constant parameters, which may be restrictive in environments characterized by policy shocks and structural breaks.

Subsequent research introduced time variation into connectedness analysis. The TVP-VAR framework [14] allows spillover structures to evolve over time without relying on arbitrary rolling windows, making it particularly suitable for analyzing markets affected by trade conflicts and other large external shocks. At the same time, connectedness analysis has been extended to the frequency domain. Baruník and Křehlík [15] decompose spillovers across different horizons, enabling a distinction between short-run fluctuations driven by market sentiment and longer-run adjustments associated with structural factors such as supply-chain reconfiguration.

Recent studies combining these approaches suggest that commodity markets exhibit both time-varying and frequency-dependent spillover dynamics, particularly under major shocks. These findings highlight the importance of jointly considering temporal evolution and horizon heterogeneity when analyzing risk transmission in globally integrated markets.

Overall, while existing studies have made important progress in understanding both trade-war effects and cross-market spillovers, there remains a lack of integrated analysis linking trade reallocation, price formation, and dynamic connectedness. To address this gap, this study employs a unified TVP-VAR framework that combines the Diebold–Yilmaz connectedness approach with frequency decomposition, allowing for a comprehensive analysis of both the evolution and persistence of spillovers in the global soybean market.

## 3. Model and Data

### 3.1. Model

In this study, both time-domain and frequency-domain connectedness are analyzed within a unified TVP-VAR framework. The DY-based approach captures the time-varying direction and intensity of spillovers, while the BK-based decomposition provides additional insights into their persistence across different horizons.

#### 3.1.1. TVP-VAR-DY Model

The TVP-VAR-DY framework combines a time-varying parameter vector autoregression (TVP-VAR) with the spillover connectedness measures of Diebold and Yılmaz [12] to characterize time-evolving interactions and risk transmission across markets. The procedure consists of the following steps.

First, construct the TVP-VAR(p) model:

(1) $$y_{t}=c_{t}+B_{1,t}y_{t-1}+\cdots +B_{p,t}y_{t-p}+\epsilon_{t}=X_{t}\beta_{t}+\epsilon_{t},\epsilon_{t}∼N\left(0,\Omega_{t}\right)$$

where, $y_{t}$ is a K×1 vector of endogenous variables; $X_{t}=I_{K}⊗\left[1,y_{t-1}^{\prime },\ldots ,y_{t-p}^{\prime }\right]$ is a K×n matrix (n=K(pK+1)), with ⊗ denoting the Kronecker product; $\beta_{t}$ is an n×1 vector stacking all time-varying coefficients (intercept $c_{t}$ and $B_{i,t}$); $\Omega_{t}$ is a K×K time-varying covariance matrix.

The time-varying parameter vector $\beta_{t}$ is assumed to follow a first-order random walk process, tracked by the Kalman filter:

(2) $$\beta_{t}=\beta_{t-1}+\nu_{t},\nu_{t}∼N\left(0,Q_{t}\right)$$

The time-varying covariance matrix $\Omega_{t}$ is decomposed to model its evolution:

(3) $$\Omega_{t}=A_{t}^{-1}H_{t}{\left(A_{t}^{-1}\right)}^{\prime }$$

Here, $A_{t}$ is a lower triangular matrix (with ones on the diagonal) capturing time-varying contemporaneous relations, and $H_{t}=diag\left(h_{1,t},\ldots ,h_{K,t}\right)$ represents time-varying stochastic volatilities. The non-zero, non-diagonal elements of $A_{t}$ (stacked as $\alpha_{t}$) and $h_{t}=ln\left(diag\left(H_{t}\right)\right)$ are also assumed to follow random walk processes.

The state-space model is estimated using the Kalman Filter recursion, enhanced with a forgetting factor λ and a decay factor κ (both typically set just below 1, e.g., 0.99) to control the rate of parameter evolution and handle time-varying variance efficiently.

The spillover effect is calculated based on Generalized Forecast Error Variance Decomposition (GFEVD).

For the filtered parameter set ($\beta_{t},\Omega_{t}$) at each time t, we treat it as a static VAR and compute the H-step-ahead generalized variance decomposition:

(4) $$\theta_{ij,t}^{g}(H)=\frac{\sigma_{jj,t}^{-1}\sum_{h=0}^{H-1}\;\;{\left(e_{i}^{\prime }\Phi_{h,t}\Omega_{t}e_{j}\right)}^{2}}{\sum_{h=0}^{H-1}\;\;\left(e_{i}^{\prime }\Phi_{h,t}\Omega_{t}\Phi_{h,t}^{\prime }e_{i}\right)}$$

where $\Phi_{h,t}$ is the h-th coefficient matrix from the VMA(∞) representation at time $t,\sigma_{jj,t}$ is the j-th diagonal element of $\Omega_{t}$, and $e_{i}$ is a selection vector.

The contributions are normalized by row to obtain proportional shares:

(5) $${\tilde{\theta }}_{ij,t}^{g}(H)=\frac{\theta_{ij,t}^{g}(H)}{\sum_{j=1}^{K}\;\;\theta_{ij,t}^{g}(H)}$$

Based on the normalized matrix ${\tilde{\Theta }}_{t}^{g}(H)=\left[{\tilde{\theta }}_{ij,t}^{g}(H)\right]$, the following indices are computed for each time t:

Total Spillover Index:

(6) $$T{SI}_{t}=\frac{\sum_{i,j=1,i\neq j}^{K}\;\;{\tilde{\theta }}_{ij,t}^{g}(H)}{K}\times 100$$

Directional Spillover Indices (for variable i):

Spillovers Transmitted (TO others):

(7) $$DS_{i\to \cdot ,t}=\sum_{j=1,j\neq i}^{K}\;{\tilde{\theta }}_{ji,t}^{g}\left(H\right)$$

Spillovers Received (FROM others):

(8) $$DS_{i\leftarrow \cdot ,t}=\sum_{j=1,j\neq i}^{K}\;{\tilde{\theta }}_{ij,t}^{g}\left(H\right)$$

Net Spillover Index:

(9) $$NS_{i,t}=DS_{i\to \cdot ,t}-DS_{i\leftarrow \cdot ,t}$$

Net Pairwise Spillover Index:

(10) $$NPS_{i\to j,t}={\tilde{\theta }}_{ji,t}^{g}(H)-{\tilde{\theta }}_{ij,t}^{g}(H)$$

#### 3.1.2. TVP-VAR-BK Model

Building on the TVP-VAR framework, we further follow Ellington and Baruník [16] and employ the TVP-VAR-BK model to uncover frequency-specific dynamic connectedness across markets. This approach extends the Baruník and Křehlík [15] frequency-domain connectedness measures to a time-varying setting, allowing the total spillover to be decomposed into distinct frequency bands (e.g., short-, medium-, and long-run components). The construction proceeds as follows:

Identical to the first step of the TVP-VAR-DY model, establish a TVP-VAR(p) and obtain the timevarying coefficient matrices $\left\{B_{h,t}\right\}$ and covariance matrix $\Sigma_{t}$ for each time point t, via Kalman Filter.

At a given time t, the model is treated as a “quasi-static” VAR. Compute its frequency response function $\Psi_{t}\left(e^{-i\omega }\right)$, the Fourier transform of the time-varying MA coefficients:

(11) $$\Psi_{t}\left(e^{-i\omega }\right)=\sum_{h=0}^{\infty }\;e^{-i\omega h}B_{h,t}\;$$

where $i=\sqrt{-1}$, and ω is frequency ∈(−π,π).

Then, compute the time-varying causal spectral density matrix $f_{t}(\omega )$:

(12) $$f_{t}(\omega )=\frac{1}{2\pi }\Psi_{t}\left(e^{-i\omega }\right)\Sigma_{t}\Psi_{t}^{\prime }\left(e^{+i\omega }\right)$$

Baruník and Křehlík [15] define the contribution of shock k to the forecast variance of variable j within a specific frequency band d=(a,b) (e.g., short-term: 1–5 days, band $d_{S}$), denoted as $\phi_{jk,t}(d)$.

First, define the power spectrum weighting function $W_{j,t}(\omega )$ for variable j at frequency ω:

(13) $$W_{j,t}(\omega )=\frac{e_{j}^{\prime }\Psi_{t}\left(e^{-i\omega }\right)\Sigma_{t}\Psi_{t}^{\prime }\left(e^{+i\omega }\right)e_{j}}{\frac{1}{2\pi }\int_{-\pi }^{\pi }\;\;e_{j}^{\prime }\Psi_{t}\left(e^{-i\lambda }\right)\Sigma_{t}\Psi_{t}^{\prime }\left(e^{+i\lambda }\right)e_{j}d\lambda }$$

Then, compute the Generalized Variance Decomposition (GFEVD) for band d:

(14) $$\phi_{jk,t}(d)=\frac{\frac{1}{2\pi }\int_{\omega \in d}\;\;W_{j,t}(\omega ){\left(f_{t}(\omega )\right)}_{jk}d\omega }{\sum_{k=1}^{N}\;\;\phi_{jk,t}(\infty )}$$

where $\phi_{jk,t}(\infty )=\frac{1}{2\pi }\int_{-\pi }^{\pi }\;W_{j,t}(\omega ){\left(f_{t}(\omega )\right)}_{jk}d\omega$ is the full-frequency contribution used for normalization, ensuring $\sum_{k=1}^{N}\;\phi_{jk,t}(d)=1$.

Using the normalized band-wise GFEVD ${\tilde{\phi }}_{jk,t}(d)=\phi_{jk,t}(d)$, we construct:

Frequency Band Total Spillover Index (within band d):

(15) $$T{SI}_{t}(d)=\left(1-\frac{Tr\left\{{\tilde{\Phi }}_{t}(d)\right\}}{\sum_{j=1}^{N}\;\;\sum_{k=1}^{N}\;\;{\tilde{\phi }}_{jk,t}(d)}\right)\times 100$$

Frequency Band Directional Spillover Indices:

From variable j to all others in band d:

(16) $$TO_{j\to \cdot ,t}(d)=\left(\frac{\sum_{k=1,k\neq j}^{N}\;\;{\tilde{\phi }}_{kj,t}(d)}{\sum_{j=1}^{N}\;\;\sum_{k=1}^{N}\;\;{\tilde{\phi }}_{jk,t}(d)}\right)\times 100$$

To variable j from all others in band d:

(17) $${FROM}_{j\leftarrow \cdot ,t}(d)=\left(\frac{\sum_{k=1,k\neq j}^{N}\;\;{\tilde{\phi }}_{jk,t}(d)}{\sum_{j=1}^{N}\;\;\sum_{k=1}^{N}\;\;{\tilde{\phi }}_{jk,t}(d)}\right)\times 100$$

Frequency Band Net Spillover Index:

(18) $$NET_{j,t}(d)=TO_{j\to \cdot ,t}(d)-FROM_{j\leftarrow \cdot ,t}\left(d\right)$$

Frequency Band Net Pairwise Directional Spillover Index:

(19) $$NPD_{j\to k,t}(d)=\left(\frac{{\tilde{\phi }}_{kj,t}(d)-{\tilde{\phi }}_{jk,t}(d)}{\sum_{j=1}^{N}\;\;\sum_{k=1}^{N}\;\;{\tilde{\phi }}_{jk,t}(d)}\right)\times 100$$

### 3.2. Data

This study examines how trade frictions reshape the global soybean pricing system. China is by far the world’s largest soybean importer and accounts for roughly two-thirds of global soybean import demand in recent years, implying that China’s futures and import prices may exert a disproportionate influence on international price discovery and cross-market price transmission [17]. Accordingly, we construct our empirical system using the following price series: (i) the Chicago Board of Trade soybean futures price *CBOT_Soybean*, widely regarded as the benchmark for global soybean pricing; (ii) the Dalian Commodity Exchange soybean futures price *DCE_Soybean*, proxied by the most-active contract of No. 1 Yellow Soybean; (iii) China’s CNF (cost-and-freight) import quotation for U.S.-origin soybeans at the U.S. Gulf *CNF_US_Gulf*, a core export corridor through which about 60% of U.S. soybean exports are shipped [18]; (iv) China’s CNF import quotation for Brazilian-origin soybeans *CNF_Brazil*; and (v) China’s CNF import quotation for Argentine-origin soybeans *CNF_Argentina*. Brazil, the United States, and Argentina are the three largest soybean producers globally and constitute China’s most important import origins; hence, the corresponding CNF series provide a representative measure of China’s import costs and the external price signals embedded in the global soybean trade.

Our dataset consists of daily observations from 6 January 2015 to 24 November 2025. The sample spans two episodes of Sino–U.S. trade frictions and covers several major global shocks, including the COVID-19 pandemic and the Russia–Ukraine war. After excluding non-trading days and removing observations with missing values, the final sample contains 2426 daily observations. All series are obtained from the Wind database.

**Figure 1:**
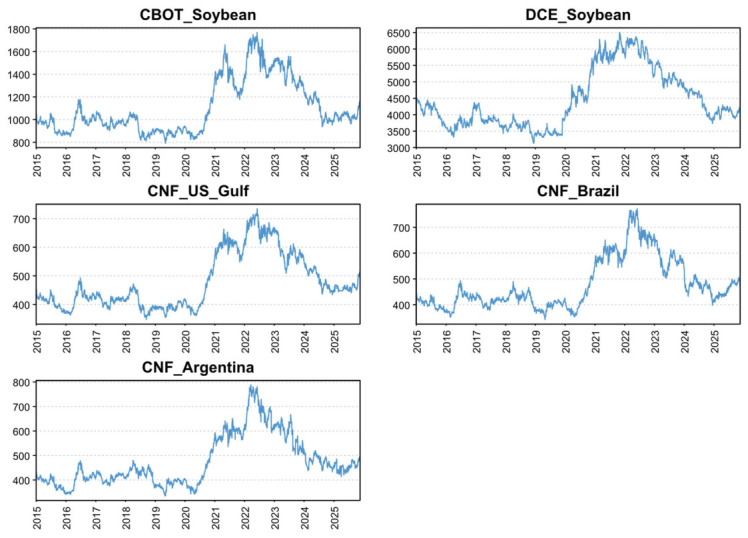
Trends in the variables over the sample period (2015–2025). Note: CBOT = Chicago Board of Trade soybean futures; DCE = Dalian Commodity Exchange soybean futures; CNF = Cost and Freight prices.

Figure 1 plots the sample paths of the variables. Overall, the five price series co-move closely, exhibiting broadly consistent dynamics over the sample period. During 2015–2019, global soybean prices remained subdued, consistent with heightened uncertainty and disruptions associated with the Sino–U.S. trade war. From 2020 to 2022, prices rose persistently and reached historical highs, reflecting strong post-pandemic demand alongside supply-chain disruptions to global grain markets following the Russia–Ukraine conflict. After 2023, price increases moderated and volatility intensified. In addition, Brazil’s expansion in soybean exports, overtaking the United States as the largest exporter in the post-trade-frictions period, exerted downward pressure on CBOT prices, contributing to a pattern of oscillation and gradual correction. Notably, China’s CNF import prices from the three source countries track CBOT closely, as these import prices are typically quoted as CBOT-based contracts with origin-specific premiums/discounts.

**Figure 2:**
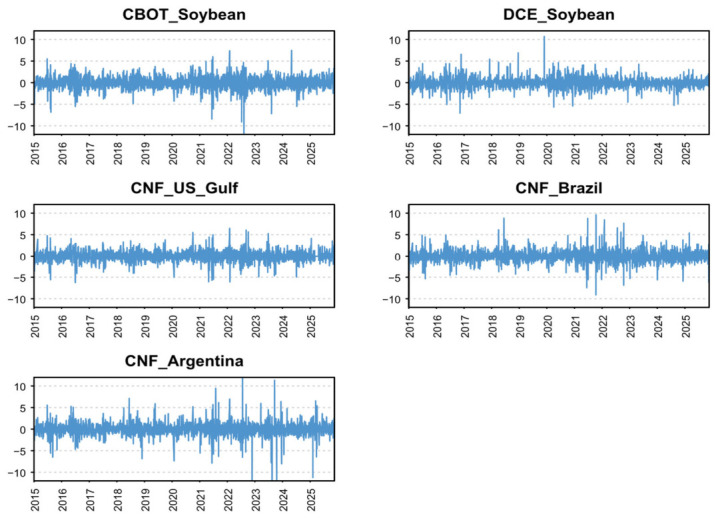
Fluctuations in the variables measured by log differences (2015−2025). Note: CBOT = Chicago Board of Trade soybean futures; DCE = Dalian Commodity Exchange soybean futures; CNF = Cost and Freight prices.

To mitigate scale effects and improve stationarity, we follow Cao and Cheng [19] and Xue et al. [20] and apply log-differencing to all variables. The resulting series can be interpreted as percentage changes (i.e., returns). Figure 2 reports these transformed series.

Table 1 reports descriptive statistics for the log-differenced series (i.e., daily returns). Notably, *DCE_Soybean* is the only market with a negative mean return, suggesting that the Sino–U.S. trade war exerted downward pressure on China’s domestic soybean futures market. *CNF_Argentina* exhibits the highest mean return but also the largest standard deviation, consistent with a risk-return trade-off. *CNF_US_Gulf* also shows a relatively high mean, suggesting that U.S. soybeans remained competitive in the international market over the sample period.

In terms of distributional properties, skewness and kurtosis, together with the Jarque-Bera (JB) test, indicate that all series are leptokurtic (fat-tailed) and deviate from normality. *DCE_Soybean* and *CNF_Brazil* are positively skewed, implying a higher likelihood of large positive returns relative to large negative ones, whereas *CBOT_Soybean*, *CNF_US_Gulf*, and *CNF_Argentina* are negatively skewed, pointing to comparatively greater downside tail risk. The ERS unit-root test results suggest that all return series are stationary. Ljung-Box tests up to 20 lags show evidence of serial correlation in returns for most variables, motivating the inclusion of lag dynamics. Moreover, the Ljung-Box statistics for squared returns (Q^2^(20)) strongly support volatility clustering, indicating time-varying conditional heteroskedasticity and supporting the use of a time-varying modeling framework.

## 4. Result

### 4.1. Time-Domain Spillover Analysis Using the TVP-VAR-DY Model

We first employ the TVP-VAR-DY framework to examine both static and dynamic connectedness among the soybean price series. Prior to estimation, the lag length is selected based on standard information criteria (Table 2). As reported in Table 2, the AIC, BIC, and SBIC consistently indicate an optimal lag order of one. Following the connectedness literature, we set the forecast-error variance decomposition (FEVD) horizon to 20 steps. The main model specifications and parameter settings are summarized in Table A1 of Appendix A.

#### 4.1.1. Static Risk Spillovers

Table 3 reports the static risk spillovers results. Each row summarizes the risk spillovers received by a given variable from all other variables, while each column summarizes the risk spillovers transmitted by that variable to the rest of the system. The “FROM” column reports the total risk spillovers received by each variable, the “TO” row reports the total risk spillovers transmitted, and the “NET” row reports net risk spillovers (transmitted minus received). The TSI denotes the total spillover index, capturing the overall degree of connectedness in the system.

The total spillover index (TSI) equals 39.83%, indicating that nearly 40% of price fluctuations in the soybean price system are attributable to cross-market shocks and risk spillovers, rather than idiosyncratic movements. This suggests that the global soybean market is highly integrated, with a moderately strong degree of interconnectedness.

*CBOT_Soybean* exhibits a net spillover of 100.23 and a high own-variance share (88.76%), implying that CBOT occupies a dominant position in the global soybean pricing system and acts as a net transmitter of risk. By contrast, *DCE_Soybean* shows a small negative net spillover (−4.47%) and a high own-variance share (87.69%), consistent with a degree of policy-driven segmentation and partial insulation in China’s domestic soybean market [21]. While domestic futures prices are exposed to international price movements, they appear to be shaped more strongly by domestic policy adjustments and other internal factors.

For the three CNF import price series, all spot markets are net receivers of risk spillovers. In particular, *CNF_US_Gulf* has a net spillover of −37.50%, and receives substantial risk spillovers from *CBOT_Soybean* (41.14%), highlighting the strong dependence of export spot prices on the CBOT futures benchmark. Similarly, *CNF_Brazil* and *CNF_Argentina* also depend heavily on CBOT prices, while bilateral spillovers between the two are non-negligible: *CNF_Brazil* transmits 12.89% to *CNF_Argentina*, and *CNF_Argentina* transmits 13.71% to *CNF_Brazil*. Overall, these results reinforce the role of CBOT as the central hub of global soybean-market risk transmission [22].

#### 4.1.2. Dynamic Risk Spillovers

Static spillover measures only capture the average intensity of interconnectedness among prices over the full sample period. To assess how the two episodes of trade frictions affect network risk spillovers over time, we therefore employ a time-varying (dynamic) spillover framework.

**Figure 3:**
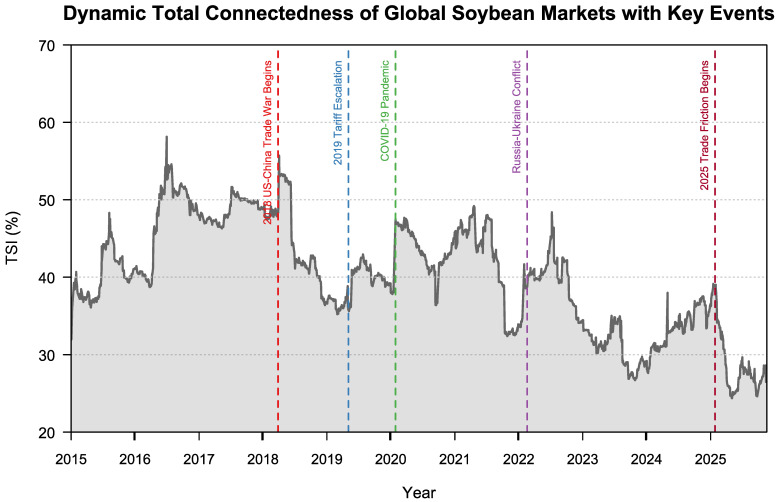
Dynamic risk spillovers and extreme events. Note: TSI = Total Spillover Index.

Figure 3 plots the evolution of the TSI. Several extreme events, namely the 2018 Sino–U.S. trade frictions, the 2019 escalation, the COVID-19 outbreak in 2020, and the Russia–Ukraine conflict in 2022, are all associated with a sharp increase in system-wide TSI. This pattern suggests that heightened uncertainty at the onset of major shocks can accelerate the transmission of risk across the entire network, consistent with Lu et al. [23], Nasir et al. [24], Ouyang et al. [25], and Cui and Maghyereh [26]. Among these episodes, the COVID-19 shock generates the largest surge in TSI, indicating that this global systemic event immediately pushed cross-market connectedness to an extreme level, exceeding the impact of other shocks [27].

By contrast, the new round of Sino–U.S. trade frictions in 2025 displays a markedly different pattern. Rather than triggering a further increase in the TSI, this episode is followed by a rapid decline in the index from around 34% to approximately 25%. This suggests that the 2025 trade shock no longer manifests itself primarily through system-wide risk amplification. Instead, it reflects a reorganization of the transmission mechanism and a structural reconfiguration of the global soybean price network.

Following the 2018 trade frictions, China had already begun to diversify its soybean import supply system. When bilateral trade tensions escalated again in 2025, China shifted further toward Brazilian soybean imports, and Brazil replaced the United States as China’s largest soybean supplier. This adjustment in import structure reduced China’s dependence on U.S. soybeans and, more broadly, on the CBOT-centered pricing system. As a consequence, the previously centralized risk transmission channels organized around the U.S. market were gradually weakened.

This interpretation is further supported by the dynamic net spillover and pairwise connectedness results discussed below. After 2025, the intensity of risk transmission among several key markets declines noticeably. In particular, the dominant transmission channel linking the U.S. benchmark price to China-related markets becomes substantially weaker, whereas the linkages between China and South American supply-chain markets become relatively stronger. Taken together, these changes indicate that the global soybean pricing system has gradually evolved from a highly integrated structure centered on CBOT and the U.S. market into a more regionally differentiated, multi-center network characterized by weaker cross-regional connectedness, which is ultimately reflected in the persistent decline in the TSI.

**Figure 4:**
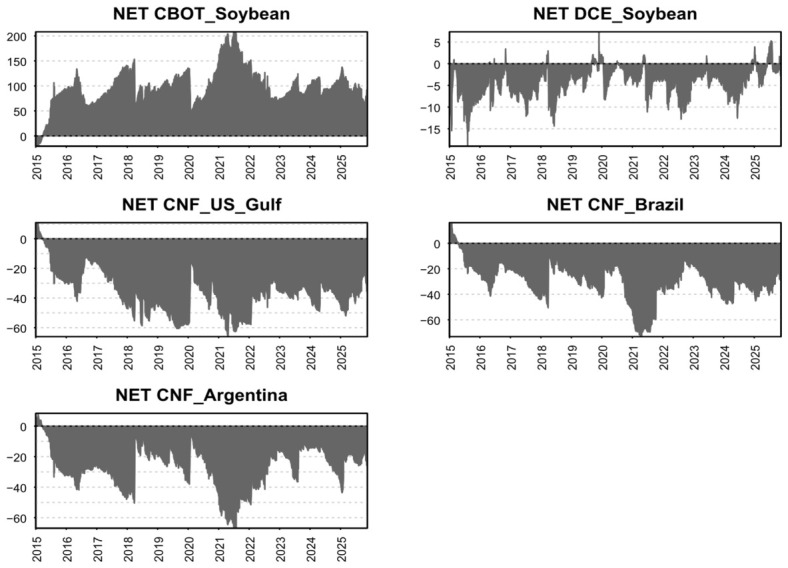
Dynamic net risk spillovers of each price series. Note: CBOT = Chicago Board of Trade soybean futures; DCE = Dalian Commodity Exchange soybean futures; CNF = Cost and Freight prices.

Figure 4 reports the time-varying net spillover measures for each price series. Over the sample period, *CBOT_Soybean* consistently acts as a net transmitter of risk, whereas *CNF_US_Gulf*, *CNF_Brazil*, and *CNF_Argentina* are persistently net receivers. Following the April 2018 Sino–U.S. trade-friction episode, when China announced a 25% tariff on U.S. soybeans, the net spillover from *CBOT_Soybean* declines sharply. At the same time, *CNF_Brazil* and *CNF_Argentina* exhibit a pronounced reduction in net risk absorption, suggesting that the dominant role of *CBOT_Soybean* weakened after the 2018 shock.

The COVID-19 outbreak in 2020 temporarily amplifies the net spillover from *CBOT_Soybean*; however, as economic conditions normalize, its net spillover reverts to its pre-shock level. After the 2025 Sino–U.S. trade-friction episode, the net spillover of *CBOT_Soybean* weakens further, while *CNF_Brazil* and *CNF_Argentina* display a further decline in net risk inflows. This pattern is consistent with the TSI results and points to a more diversified pricing structure in the global soybean market after trade frictions: CBOT’s pricing influence appears to have diminished, while Brazil and Argentina have gained prominence in China’s soybean import sourcing.

**Figure 5:**
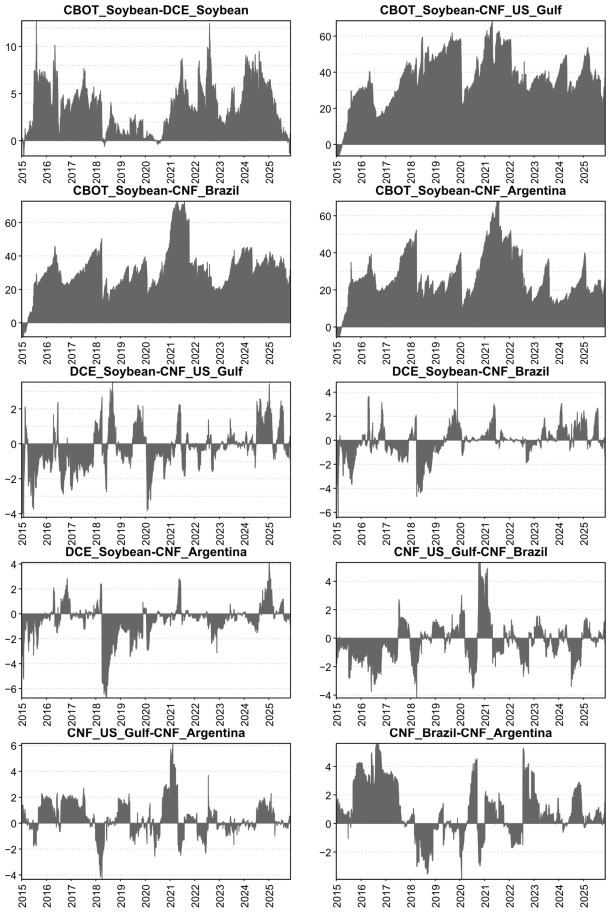
Pairwise risk spillovers among soybean prices. Note: CBOT = Chicago Board of Trade soybean futures; DCE = Dalian Commodity Exchange soybean futures; CNF = Cost and Freight prices; positive (negative) values indicate net spillovers transmitted to (received from) other markets.

Figure 5 reports the pairwise risk spillovers among soybean prices and reveals three broad patterns. First, the outward influence of *CBOT_Soybean* on China-related markets weakens after the 2018 trade-friction episode and declines further after the renewed escalation in 2025, particularly for *CNF_Brazil* and *CNF_Argentina*. By contrast, its spillover to *CNF_US_Gulf* remains comparatively more stable, indicating that while trade frictions weakened CBOT’s role in China-facing price transmission, they did not fundamentally alter the U.S.-centered pricing linkage between CBOT futures and Gulf export quotations. Second, the relationship between *CBOT_Soybean* and *DCE_Soybean* becomes increasingly less one-sided. Although CBOT remains the dominant transmitter over most of the sample, its net spillover to DCE falls substantially after 2018 and turns negative again after 2025, suggesting that China’s domestic pricing benchmark has become more influential under a more diversified import structure. Third, *DCE_Soybean* and South American CNF prices become more tightly linked over time, while within South America, *CNF_Brazil* more often acts as the short-run leader relative to *CNF_Argentina*. Taken together, these patterns point to a gradual transition from a CBOT-centered system toward a more regionally differentiated pricing structure in which China-facing demand conditions and South American export benchmarks play increasingly important roles.

### 4.2. Frequency-Domain Spillover Analysis Using the TVP-VAR-BK Model

To provide a finer-grained assessment of how price volatility risk is transmitted across different horizons, we further employ the TVP-VAR-BK framework to decompose system connectedness in the frequency domain. Following standard practice, we partition the dynamics into short-term (1–5 days), medium-term (5–22 days), and long-term (beyond 22 days) components.

#### 4.2.1. Frequency-Specific Static Spillovers

Table 4, Table 5 and Table 6 report the static connectedness of the global soybean market over the short-, medium-, and long-term horizons, respectively. In terms of the TSI, connectedness declines sharply as the horizon lengthens. Specifically, the short-term TSI reaches 30.58, indicating intense and high-frequency risk transmission across markets. At this horizon, price fluctuations are primarily driven by breaking news, high-frequency trading flows, short-lived sentiment, and arbitrage activity, such that information is rapidly disseminated and incorporated into prices within the global market network [28]. The TSI then falls markedly to 6.95 in the medium term and further to 2.54 in the long term. As the horizon extends, prices are increasingly shaped by fundamentals, such as supply conditions, long-run demand trends, and structural trade policies, leading to weaker cross-market synchronization [29]. This pattern underscores the dual nature of the soybean market: it behaves as a globally integrated financial market that is highly sensitive to real-time information in the short run, while in the longer run it resembles a commodity market governed more by region-specific supply–demand structures.

Focusing on futures prices, *CBOT_Soybean* exhibits dominant short-run price leadership. In the short term, its net spillover equals 79.86, and its spillovers to *CNF_US_Gulf*, *CNF_Brazil*, and *CNF_Argentina* are 32.64, 28.09, and 23.83, respectively. These results suggest that, as a benchmark for global soybean pricing, shocks to CBOT futures tend to generate pronounced short-run comovement across markets. However, CBOT Soybean’s net spillover declines rapidly to 17.82 in the medium term and 6.51 in the long term, with its spillovers to other markets weakening accordingly. Overall, the influence of CBOT futures appears to be concentrated in short-run financial transmission, whereas its ability to shape longer-run price dynamics diminishes substantially as regional markets revert to fundamentals-driven pricing.

In contrast to the attenuation pattern observed for *CBOT_Soybean*, the role of *DCE_Soybean* evolves in the opposite direction. In the short run, *DCE_Soybean* acts as a risk receiver, with a net spillover of −3.15. Around 8.98% of its volatility is attributable to external shocks, nearly half of which originate from CBOT, highlighting the short-run openness of China’s market and its sensitivity to global financial conditions. At medium and long horizons, however, *DCE_Soybean*’s net risk absorption declines markedly and moves close to balance, with its fluctuations increasingly driven by idiosyncratic factors. This suggests a degree of relative pricing autonomy for *DCE_Soybean*, particularly at horizons beyond one month, where dynamics are more closely tied to China’s domestic supply–demand conditions, macroeconomic policies, and longer-term trading arrangements with South American exporters.

Turning to CNF prices, *CNF_US_Gulf* exhibits a pronounced short-run reliance on financial transmission. Its received spillovers amount to 47.18% in the short term, with the majority stemming from *CBOT_Soybean* (32.64%), and its net spillover is −29.46%. This pattern is consistent with its role as the nearest delivery location for *CBOT_Soybean* futures: even small futures-price movements can be transmitted to spot quotations through the shortest pricing channel, generating highly financialized and information-sensitive dynamics. In the medium and long run, received spillovers fall to 11.00% and 4.02%, respectively. This indicates that, beyond the short horizon, CNF pricing is shaped less by transitory futures fluctuations and more by real-economy determinants, including inland logistics constraints, exporters’ inventory positions, contract-specific terms, and relative competitiveness vis-à-vis other origins. Accordingly, local supply–demand conditions and trade structures become increasingly important for the formation of forward premia/discounts, weakening the dominance of financial-market impulses.

Evidence from South America further reinforces, and refines, our earlier conclusions regarding regional structural features. In the short term, *CNF_Brazil* exerts a notable one-way spillover to *CNF_Argentina*, with a net spillover of approximately −0.64, consistent with the view that Brazil serves as the regional price leader: in immediate trading conditions, Brazilian quotes often function as a benchmark within South America, and their movements can quickly propagate to Argentine spot prices. At medium and long horizons, spillovers between the two markets are weak and become more balanced in direction. A plausible explanation is that the long-run anchors of soybean pricing differ substantially across the two countries. Brazilian prices are primarily driven by domestic fundamentals such as production costs, output scale, and longer-term contracts with China [30,31], whereas Argentine prices depend more on domestic monetary conditions, export tax regimes, and farmers’ storage and marketing behavior [32]. As a result, longer-run dynamics evolve around distinct institutional settings and macroeconomic environments. Overall, short-run comovement mainly reflects common exposure to external shocks, most notably shifts in China’s procurement pace, whereas long-run divergence is rooted in country-specific policy frameworks and macroeconomic conditions, yielding a pattern of short-run integration but structural separation over longer horizons.

#### 4.2.2. Frequency-Specific Dynamic Spillovers

Next, we examine the dynamic spillovers across frequency bands. In the figure, the orange, green, and purple lines correspond to short-term, medium-term, and long-term spillovers, respectively. Spillover dynamics are broadly similar across horizons, although total connectedness is predominantly driven by short-term spillovers [33].

**Figure 6:**
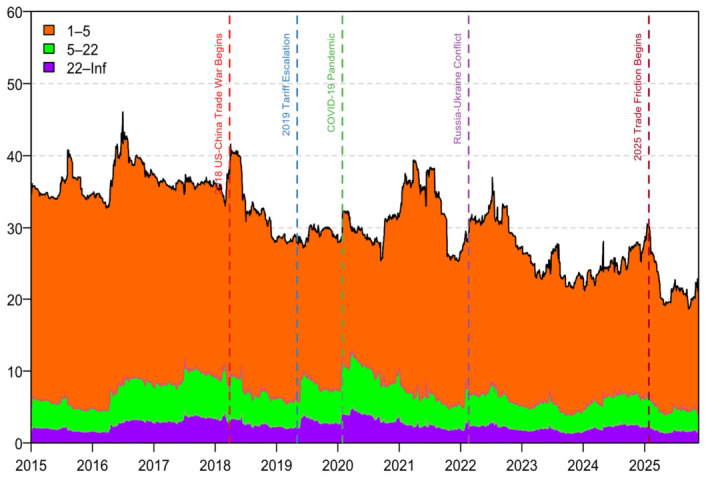
Dynamic risk spillovers across frequency bands.

One point merits emphasis. On 22 March 2018, U.S. President Donald Trump signed a memorandum under the Section 301 investigation, directing the USTR to advance additional tariff actions; on 3 April 2018, the USTR released the proposed product list, widely viewed as the starting point of the Sino–U.S. trade war. Against this backdrop, short-, medium-, and long-horizon connectedness in the soybean market all increase noticeably (Figure 6), consistent with a broad-based surge in uncertainty. During this episode, market participants, from financial traders to physical merchandisers, faced unusually opaque expectations regarding policy paths and trade responses, amplifying co-movement and risk transmission across all frequency bands.

A different pattern emerges during the subsequent escalation. On 10 May 2019, the United States raised tariffs from 10% to 25% on roughly USD 200 billion of Chinese imports. In parallel, the trade disruption reallocated demand toward South America and pushed Brazilian soybean export premia higher. Notably, short-term connectedness changes little, whereas medium- and long-term connectedness rise markedly. This suggests that the tariff escalation and the associated trade-flow interruptions were not priced as a merely transitory shock; instead, they were increasingly incorporated as a structural shift, reflecting a reassessment of medium-to-long-run supply–demand conditions under an emerging “new normal” characterized by intensified decoupling pressures and a southward shift in sourcing. Collectively, these dynamics provide suggestive evidence of supply-chain reconfiguration and a rebalancing of price leadership in global soybean pricing.

**Figure 7:**
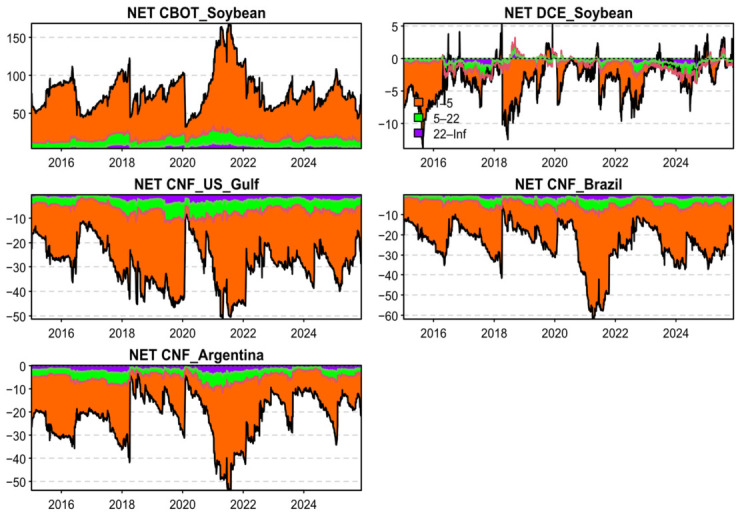
Dynamic net risk spillovers across frequency bands. Note: CBOT = Chicago Board of Trade soybean futures; DCE = Dalian Commodity Exchange soybean futures; CNF = Cost and Freight prices.

Figure 7 presents the frequency-specific dynamic net spillovers for each price series. Consistent with the patterns in overall connectedness, the time variation in net spillovers is predominantly driven by the short-run band. Following the escalation of trade frictions in May 2019, *CBOT_Soybean*’s short-run net spillover declines, while its medium- and long-run net spillovers increase slightly. This pattern is consistent with the idea that the physical disruption of U.S. soybean exports to China weakened the high-frequency financial channel linking CBOT price fluctuations to China’s immediate demand conditions, even though CBOT continued to retain influence in global soybean price discovery.

In contrast, *DCE_Soybean*’s net spillover switches from negative to positive, and this tendency becomes more pronounced after 2025, suggesting that fluctuations in China’s soybean futures increasingly shape spot-market trade flows and pricing expectations, implying a strengthening of China’s pricing influence. Around 2024, *CNF_Brazil* becomes more negative while *CNF_Argentina* becomes less negative, indicating a redistribution of pricing influence within the South American supply chain. On the one hand, as Brazil remains China’s largest and most stable supplier, its pricing appears to exhibit stronger one-sided sensitivity to changes in Chinese demand, leading to a rising net exposure to external demand shocks. On the other hand, Argentina, often serving as a key alternative origin, appears to gain greater pricing autonomy and bargaining power amid China’s strategy of import diversification and supply-chain security, thereby reducing its net exposure to external financial and demand shocks.

After the renewed Sino–U.S. trade frictions in 2025, *CBOT_Soybean*’s net spillover weakens further, and *CNF_Brazil* and *CNF_Argentina* also exhibit a further decline in risk absorption from the system. Taken together, these results suggest the gradual emergence of a more regionalized pricing architecture, centered on Chinese demand, with DCE futures and South American CNF spot benchmarks playing increasingly important roles.

**Figure 8:**
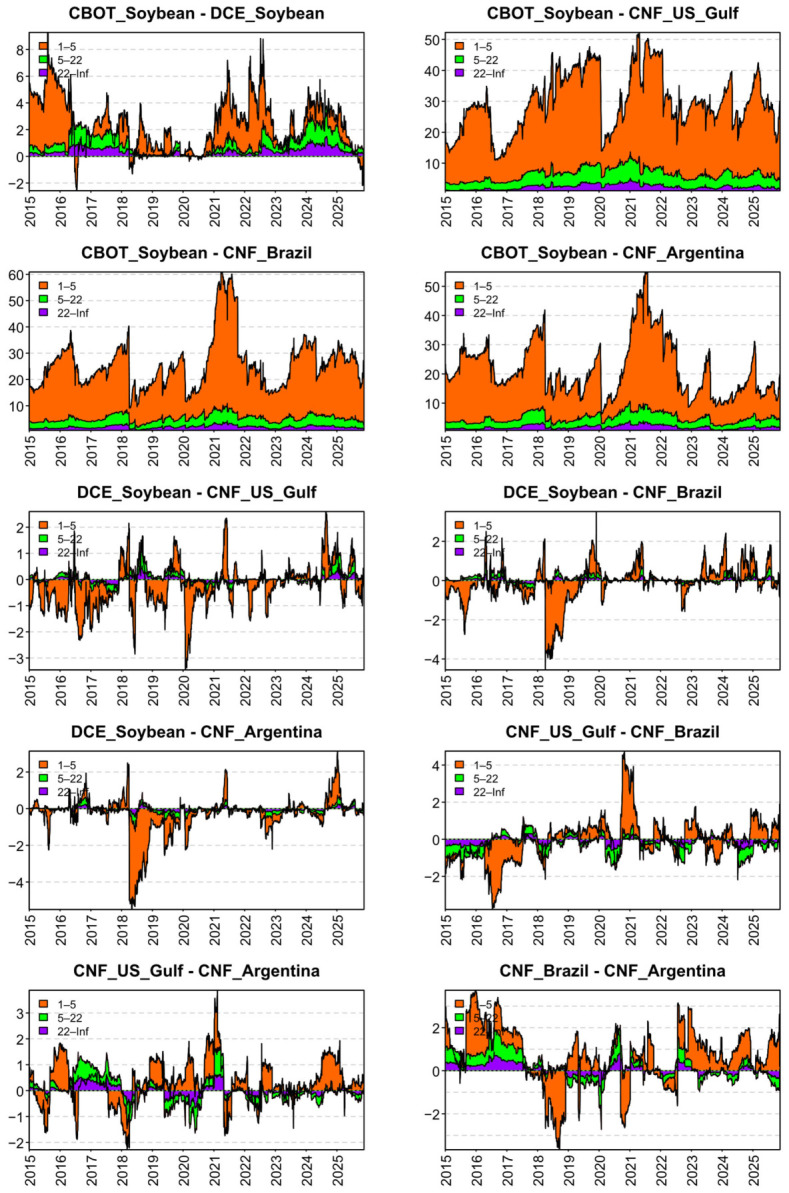
Pairwise network risk spillovers across frequency bands. Note: CBOT = Chicago Board of Trade soybean futures; DCE = Dalian Commodity Exchange soybean futures; CNF = Cost and Freight prices; positive (negative) values indicate net spillovers transmitted to (received from) other markets.

Figure 8 presents the pairwise connectedness across frequency bands and provides further evidence of a reconfiguration of pricing influence in the global soybean market. Overall, the frequency-domain results confirm that *CBOT_Soybean* remains the dominant short-run transmitter, but its influence on China-facing markets weakens substantially after the trade-friction episodes, especially for *CNF_Brazil* and *CNF_Argentina*. By contrast, *DCE_Soybean* becomes increasingly important in medium- and short-run spillovers to South American CNF prices, indicating that China’s demand-side pricing signals are becoming more embedded in regional export quotations.

Within South America, the linkage between *CNF_Brazil* and *CNF_Argentina* is strongly horizon-dependent. *CNF_Brazil* generally leads in the short run, although this relationship temporarily reverses during the 2018–2019 trade-friction period. After the 2025 escalation, *CNF_Brazil* acts as a short-run transmitter but becomes a medium- and long-run receiver, suggesting that short-term regional pricing remains anchored by Brazil, whereas longer-run dynamics are more influenced by Argentina’s policy-related risks.

Overall, the frequency-domain evidence suggests that the soybean pricing system has shifted away from a purely CBOT-centered structure toward a more multi-centered configuration in which pricing influence is distributed across the U.S. benchmark, China’s domestic futures market, and South American export benchmarks.

## 5. Robustness Test

### 5.1. Model Specification Robustness Tests

To ensure the robustness of our findings, we assess the sensitivity of the results to key modeling choices.

**Figure 9:**
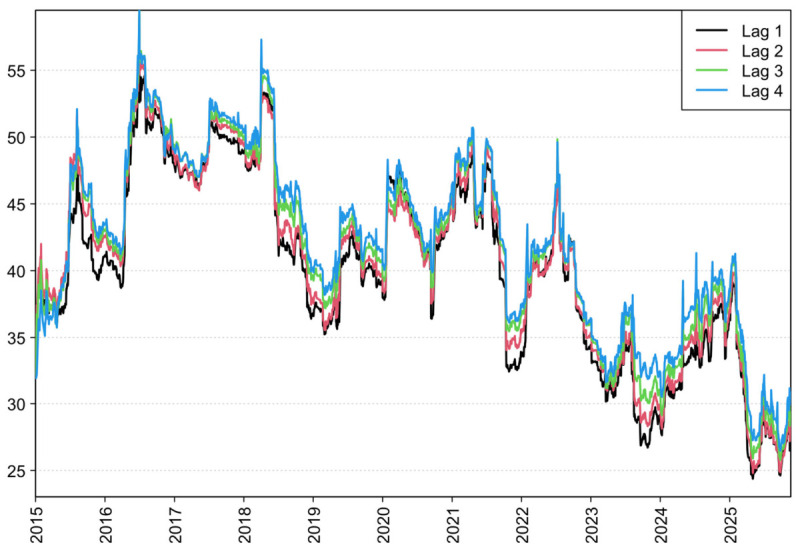
Robustness to alternative lag orders.

First, we examine robustness to the lag-order specification. In addition to the baseline TVP-VAR-DY model with one lag, we re-estimate models with two, three, and four lags, and compute the corresponding dynamic TSI. As shown in Figure 9, the TSI series derived under alternative lag orders exhibit highly consistent time-varying patterns, and the curves remain closely aligned throughout the sample. This evidence indicates that the system’s risk-transmission dynamics and overall connectedness are not unduly driven by the chosen lag length, supporting the robustness of our main conclusions along this dimension.

**Figure 10:**
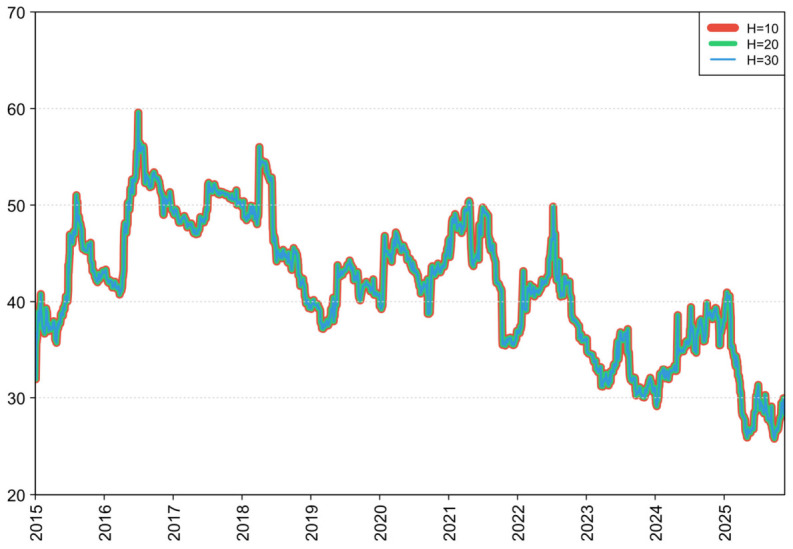
Robustness to alternative FEVD horizons.

Second, we evaluate whether the results depend on the FEVD horizon. We set the FEVD horizon to 10, 20, and 30 steps ahead and recompute the associated dynamic TSI. Figure 10 shows that the resulting index trajectories are almost indistinguishable. This finding suggests that the empirical results are largely insensitive to the choice of FEVD horizon, alleviating concerns that the conclusions are systematically affected by this parameter setting.

### 5.2. Variable and Identification Robustness Tests

#### 5.2.1. Alternative Risk Proxies

To further examine whether the conclusions on risk spillovers depend on the benchmark return specification, this study replaces the original return series with absolute returns and squared returns, and re-estimates the TVP-VAR-DY and TVP-VAR-BK models. The results, reported in Appendix A, show that the time-varying patterns of risk transmission and risk reception among the major markets, as well as the overall evolution of the global soybean price network from a single-center structure to a multi-center configuration, remain broadly unchanged. This suggests that the main conclusions are not driven by a specific return measure.

A closer comparison indicates that the dynamic total spillover levels, net spillover directions, and frequency-domain structures of most markets remain largely consistent with the benchmark results. In particular, the spillover relationships between CBOT and the CNF price series, such as *CNF_US_Gulf* and *CNF_Brazil*, change only marginally, indicating that the international soybean pricing chain anchored by CBOT remains highly stable under alternative risk proxies.

At the same time, the spillover relationship between the two core futures markets appears to be more sensitive to the definition of the risk proxy. Using absolute returns as an example, the net spillover of *DCE_Soybean* around November 2019 is substantially higher than in the benchmark specification, and the pairwise net spillover trajectory between *CBOT_Soybean* and *DCE_Soybean* differs more noticeably from the baseline results. This suggests that the benchmark model based on log returns mainly captures directional price transmission, whereas the models based on absolute or squared returns place greater emphasis on volatility intensity and risk amplification. In particular, the heightened DCE spillovers in late 2019 likely reflect intensified market uncertainty during the period of repeatedly revised expectations regarding the temporary easing of Sino–U.S. trade tensions and the resumption of U.S. soybean purchases by China.

Overall, the alternative risk proxy tests do not alter the core conclusion of this study, namely that Sino–U.S. trade frictions and the resulting restructuring of China’s import pattern have pushed the global soybean price risk network from a relatively single-pole structure centered on CBOT toward a more fragmented multi-center system. The replacement of risk proxies mainly affects the characterization of short-term volatility transmission between the core futures markets, but does not change the overall conclusions of the paper.

#### 5.2.2. Frequency-Band Specification

The identification of short-, medium-, and long-term risk spillovers in the TVP-VAR-BK model depends on the specification of the frequency bands. Based on the benchmark frequency decomposition, in which the short term is defined as 1–5 trading days, the medium term as 5–22 trading days, and the long term as more than 22 trading days, this study further implements two alternative band specifications for robustness testing. First, the boundary between the medium and long terms is extended from 22 trading days to 30 trading days, yielding a decomposition of 1–5 days, 5–30 days, and more than 30 days. Second, both the short- and medium-term boundaries are relaxed simultaneously, and the frequency bands are redefined as 1–10 days, 10–30 days, and more than 30 days. The results, reported in Appendix B, show that under different frequency-band specifications, short-term risk spillovers mainly capture market sentiment and event-driven shocks, whereas long-term risk spillovers primarily reflect changes in trade patterns and supply-chain restructuring. The main findings of this study remain stable, indicating that the frequency-domain results are robust.

#### 5.2.3. Additional Common Shock Variables

Given that the international soybean price system may be jointly affected by common external factors such as exchange rates and crude oil prices, this study further augments the baseline system by incorporating the RMB/USD exchange rate and the West Texas Intermediate (WTI) crude oil price, and then re-estimates the extended TVP-VAR model. The results, reported in Appendix C, show that after controlling for these key common shocks, the main risk spillover relationships among CBOT, DCE, and the South American CNF prices remain largely unchanged. This suggests that the reconfiguration of the soybean price risk network identified in this study is not merely driven by common exogenous shocks.

### 5.3. Excluding Extreme Event Windows

To rule out the possibility that the estimation results are driven by a small number of global extreme events, this study further conducts a robustness test by excluding extreme event windows. Considering that both the initial outbreak of COVID-19 and the early escalation of the Russia–Ukraine conflict triggered substantial turbulence in international commodity markets, the periods from 24 February 2020 to 30 April 2020 and from 24 February 2022 to 30 April 2022 are defined as extreme event windows. The TVP-VAR-DY and TVP-VAR-BK models are then re-estimated after excluding these observations. The results, reported in Appendix D, show that neither the overall evolution of the dynamic total spillover index nor the net risk-transmitting positions of the major markets changes materially. This indicates that the main conclusions of this study are not driven by a small number of extreme-event observations and are therefore robust.

## 6. Conclusions and Policy Implications

Given the central role of soybeans in feed supply and food-related processing industries, changes in the global soybean pricing system have implications not only for commodity markets but also for the resilience of broader agri-food supply chains. In recent years, repeated major external shocks, most notably the Sino–U.S. trade frictions, have challenged the CBOT-centered global soybean pricing architecture, reshaping cross-market linkages and the channels through which volatility risk is transmitted. Against this backdrop, this study employs the TVP-VAR-DY framework and its frequency-domain extension, the TVP-VAR-BK model, to analyze daily prices from 2015 to 2025 for five key benchmarks: CBOT soybean futures as the global pricing reference; DCE No. 1 soybean futures as a proxy for China’s domestic supply–demand conditions; and CNF prices capturing the landed costs of U.S., Brazilian, and Argentine soybeans delivered to China. We systematically assess how two episodes of Sino–U.S. trade frictions altered the dynamic connectedness structure of the global soybean price system.

Our results indicate that the global soybean market is highly integrated and exhibits strong time-varying interdependence. The TSI averages 39.83, with risk transmission concentrated primarily at short horizons; system-wide spillovers decline rapidly at medium and long horizons, implying that prices respond more sensitively to transitory shocks than to persistent disturbances. CBOT soybean futures remain the main source of risk spillovers, particularly in the short run, consistent with their role as the dominant global benchmark. Importantly, however, CBOT’s centrality weakens after the 2018 trade-friction episode and declines further following the 2025 episode, especially in terms of its influence on CNF prices for Brazilian and Argentine shipments to China.

Frequency-specific evidence provides additional insights. After the 2018 episode, the total spillover index moves in opposite directions across short versus medium-to-long horizons, suggesting that tariff escalations, trade-flow disruptions, and supply-chain reconfiguration were increasingly priced as structural rather than purely short-lived shocks. Around 2025, spillovers originating from DCE No. 1 soybean futures strengthen markedly, pointing to the emergence of a more regionalized pricing system centered on Chinese demand, with DCE futures and South American CNF benchmarks becoming key nodes in price discovery and expectation formation.

Within South America, a pricing configuration led by Brazil, with Argentina playing a secondary role, appears to have taken shape, particularly for short-run price formation. At medium and long horizons, however, Brazilian prices become more exposed to spillovers from Argentina’s domestic policy risk (e.g., export-tax adjustments), highlighting a complex intra-regional risk-transmission mechanism within South America’s supply network.

Building on the above findings, we derive three policy implications.

First, China should accelerate the development of an international soybean pricing hub and strengthen the external influence of the DCE. Given the rising role of DCE futures and the nascent emergence of a regional pricing mechanism, policymakers should further advance the internationalization of DCE-linked benchmarks and encourage both domestic and overseas industry participants to use DCE prices for trade pricing and risk management. A key objective is to foster a pricing chain in which Chinese demand is reflected in DCE futures prices and subsequently transmitted to regional trade quotations, thereby gradually enhancing China’s pricing power in the global soybean market.

Second, a multi-horizon supply-chain risk management framework is needed to address both short-run shocks and structural risks. Regulators and firms should explicitly account for the frequency-domain nature of market risk by establishing early-warning and response mechanisms that operate over short, medium, and long horizons. In the short run, priority should be given to monitoring high-frequency volatility and capital-flow pressures. Over medium and longer horizons, risk mitigation should focus on reducing structural vulnerabilities through import-source diversification (e.g., deeper engagement with South America and the Black Sea region), investment in overseas storage and logistics capacity, and expansion of domestic oilseed production to improve resilience against supply-chain reconfiguration.

Third, China should deepen strategic coordination with South American producers to build a more resilient regional supply network. Given the strong intra-regional pricing linkages in South America and the importance of policy-risk spillovers, a differentiated cooperation strategy is warranted. On the one hand, China should continue to strengthen long-term investment ties and stable supply arrangements with Brazil. On the other hand, greater emphasis should be placed on deepening cooperation with more “elastic” suppliers such as Argentina through flexible medium- to long-term procurement contracts, currency-swap arrangements, and supply-chain finance, which can improve adaptability to output fluctuations and policy changes. In parallel, China can leverage bilateral and multilateral platforms to encourage greater stability and predictability in South American agricultural trade policies, thereby reducing policy-driven disturbances to regional pricing and improving the stability and cost controllability of China’s soybean imports.

## Figures and Tables

**Table 1:** Descriptive statistics of the variables.

	*CBOT_Soybean*	*DCE_Soybean*	*CNF_US_Gulf*	*CNF_Brazil*	*CNF_Argentina*
Mean	0.0028	−0.0034	0.0043	0.0016	0.0049
Variance	1.8591	1.2739	1.3734	1.8079	2.1998
Skewness	−0.549 ***	0.517 ***	−0.153 ***	0.275 ***	−0.709 ***
	(0.000)	(0.000)	(0.002)	(0.000)	(0.000)
Kurtosis	6.487 ***	7.475 ***	3.816 ***	6.683 ***	16.246 ***
	(0.000)	(0.000)	(0.000)	(0.000)	(0.000)
JB	4373.814 ***	5753.740 ***	1480.726 ***	4543.856 ***	26,871.416 ***
	(0.000)	(0.000)	(0.000)	(0.000)	(0.000)
ERS	−20.801 ***	−22.223 ***	−11.046 ***	−14.875 ***	−13.064 ***
	(0.000)	(0.000)	(0.000)	(0.000)	(0.000)
Q(20)	31.720 **	12.826	25.296	37.191 **	43.248 ***
	(0.046)	(0.885)	(0.190)	(0.011)	(0.002)
Q^2^(20)	108.122 ***	22.364 ***	110.767 ***	87.899 ***	19.892 **
	(0.000)	(0.006)	(0.000)	(0.000)	(0.018)

Notes: The variables are expressed as log differences (daily returns). JB denotes the Jarque-Bera normality test. Q(20) and Q^2^(20) denote Ljung-Box statistics for returns and squared returns up to 20 lags, respectively. ERS denotes the Elliott-Rothenberg-Stock unit-root test. CBOT = Chicago Board of Trade soybean futures; DCE = Dalian Commodity Exchange soybean futures; CNF = Cost and Freight prices. ** and *** indicate statistical significance at the 5% and 1% levels, respectively.

**Table 2:** Lag-length selection.

Lag	LL	LR	df	*p*	FPE	AIC	HQIC	SBIC
0	−2258.16	1.92	14.84	14.86	14.90			
1	−2038.26	439.8 *	25.00	0.00	0.53 *	13.56 *	13.71 *	13.93 *
2	−2028.07	20.38	25.00	0.73	0.59	13.66	13.93	14.33
3	−2011.79	32.56	25.00	0.14	0.62	13.72	14.11	14.69
4	−1997.48	28.63	25.00	0.28	0.67	13.79	14.30	15.07

Notes: LL denotes the log-likelihood value; LR is the likelihood ratio test statistic; df represents degrees of freedom; *p* denotes the *p*-value; FPE is the final prediction error; AIC is the Akaike information criterion; HQIC is the Hannan–Quinn information criterion; SBIC is the Schwarz Bayesian information criterion. An asterisk (*) indicates the optimal lag order selected by the corresponding criterion.

**Table 3:** Static risk spillovers.

	*CBOT_Soybean*	*DCE_Soybean*	*CNF_US_Gulf*	*CNF_Brazil*	*CNF_Argentina*	FROM
*CBOT_Soybean*	88.76	2.22	3.30	2.67	3.05	11.24
*DCE_Soybean*	5.75	87.69	2.04	2.18	2.34	12.31
*CNF_US_Gulf*	41.14	1.79	38.28	10.66	8.14	61.72
*CNF_Brazil*	34.61	2.07	10.39	40.05	12.89	59.95
*CNF_Argentina*	29.97	1.77	8.49	13.71	46.06	53.94
TO	111.47	7.84	24.22	29.22	26.41	TSI
NET	100.23	−4.47	−37.50	−30.73	−27.53	39.83

Note: CBOT = Chicago Board of Trade soybean futures; DCE = Dalian Commodity Exchange soybean futures; CNF = Cost and Freight prices; TSI = Total Spillover Index. “From” and “To” denote directional spillovers received from and transmitted to other markets, respectively.

**Table 4:** Short-term risk spillovers (1–5 days).

	*CBOT_Soybean*	*DCE_Soybean*	*CNF_US_Gulf*	*CNF_Brazil*	*CNF_Argentina*	FROM
*CBOT_Soybean*	71.32	1.87	2.61	2.16	2.29	8.93
*DCE_Soybean*	4.23	70.18	1.54	1.58	1.63	8.98
*CNF_US_Gulf*	32.64	1.28	29.94	7.61	5.65	47.18
*CNF_Brazil*	28.09	1.45	7.60	31.32	9.49	46.63
*CNF_Argentina*	23.83	1.24	5.96	10.13	36.16	41.16
TO	88.80	5.83	17.72	21.49	19.06	TSI
Net	79.86	−3.15	−29.46	−25.14	−22.10	30.58

Note: CBOT = Chicago Board of Trade soybean futures; DCE = Dalian Commodity Exchange soybean futures; CNF = Cost and Freight prices; TSI = Total Spillover Index. “From” and “To” denote directional spillovers received from and transmitted to other markets, respectively.

**Table 5:** Medium-term risk spillovers (5–22 days).

	*CBOT_Soybean*	*DCE_Soybean*	*CNF_US_Gulf*	*CNF_Brazil*	*CNF_Argentina*	FROM
*CBOT_Soybean*	12.90	0.25	0.47	0.41	0.47	1.61
*DCE_Soybean*	1.23	13.11	0.28	0.32	0.40	2.22
*CNF_US_Gulf*	7.01	0.33	5.78	2.09	1.56	11.00
*CNF_Brazil*	5.75	0.40	1.81	5.99	2.22	10.18
*CNF_Argentina*	5.43	0.33	1.57	2.42	6.86	9.76
TO	19.43	1.31	4.13	5.24	4.65	TSI
Net	17.82	−0.92	−6.87	−4.93	−5.10	6.95

Note: CBOT = Chicago Board of Trade soybean futures; DCE = Dalian Commodity Exchange soybean futures; CNF = Cost and Freight prices; TSI = Total Spillover Index. “From” and “To” denote directional spillovers received from and transmitted to other markets, respectively.

**Table 6:** Long-term risk spillovers (beyond 22 days).

	*CBOT_Soybean*	*DCE_Soybean*	*CNF_US_Gulf*	*CNF_Brazil*	*CNF_Argentina*	FROM
*CBOT_Soybean*	4.66	0.09	0.17	0.15	0.17	0.59
*DCE_Soybean*	0.45	4.70	0.10	0.11	0.14	0.81
*CNF_US_Gulf*	2.56	0.12	2.09	0.77	0.58	4.02
*CNF_Brazil*	2.09	0.14	0.66	2.17	0.82	3.71
*CNF_Argentina*	1.99	0.12	0.58	0.89	2.48	3.57
TO	7.09	0.47	1.50	1.92	1.71	TSI
Net	6.51	−0.34	−2.52	−1.79	−1.86	2.54

Note: CBOT = Chicago Board of Trade soybean futures; DCE = Dalian Commodity Exchange soybean futures; CNF = Cost and Freight prices; TSI = Total Spillover Index. “From” and “To” denote directional spillovers received from and transmitted to other markets, respectively.

**Figure A1:**
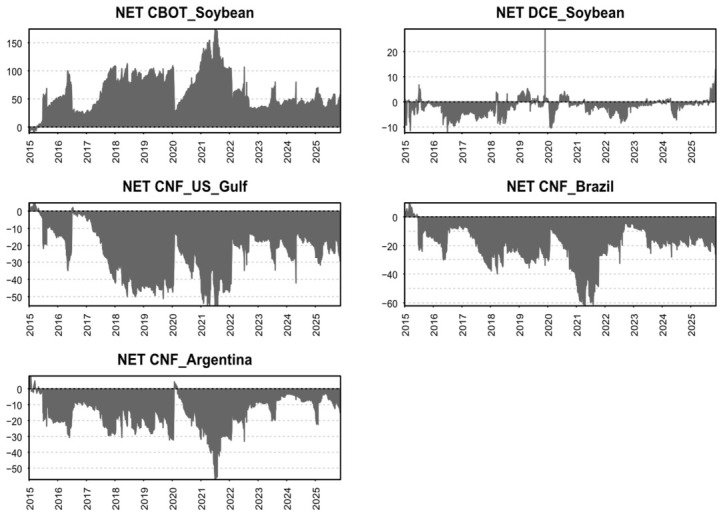
Dynamic net risk spillovers of each price series (absolute returns). Note: CBOT = Chicago Board of Trade soybean futures; DCE = Dalian Commodity Exchange soybean futures; CNF = Cost and Freight prices.

**Figure A2:**
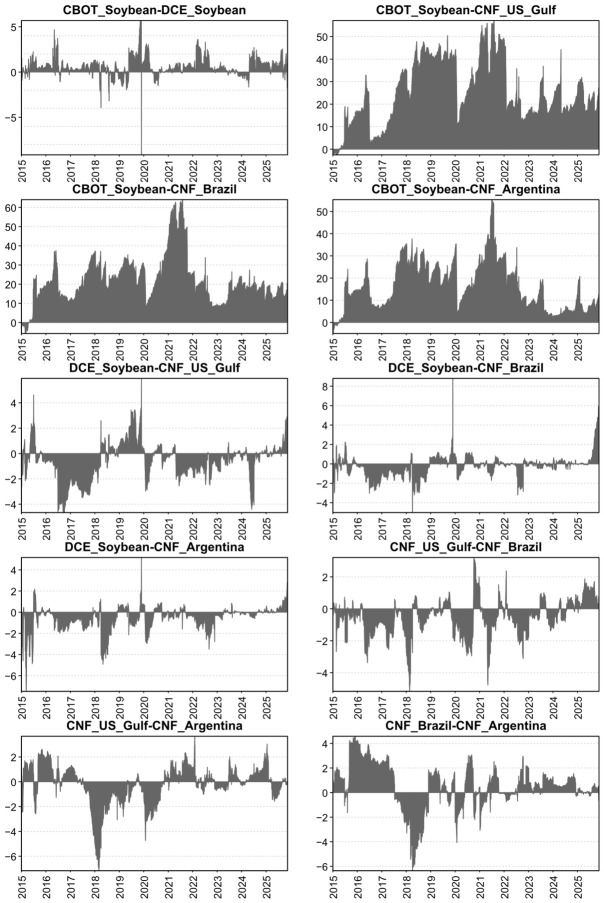
Pairwise risk spillovers among soybean prices (absolute returns). Note: CBOT = Chicago Board of Trade soybean futures; DCE = Dalian Commodity Exchange soybean futures; CNF = Cost and Freight prices; positive (negative) values indicate net spillovers transmitted to (received from) other markets.

**Figure A3:**
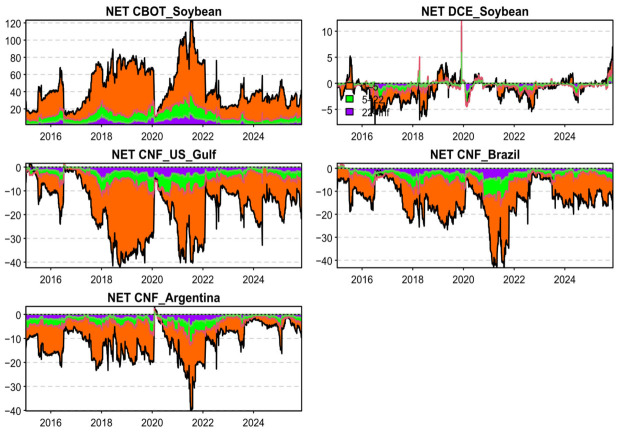
Dynamic net risk spillovers across frequency bands (absolute returns). Note: CBOT = Chicago Board of Trade soybean futures; DCE = Dalian Commodity Exchange soybean futures; CNF = Cost and Freight prices.

**Figure A4:**
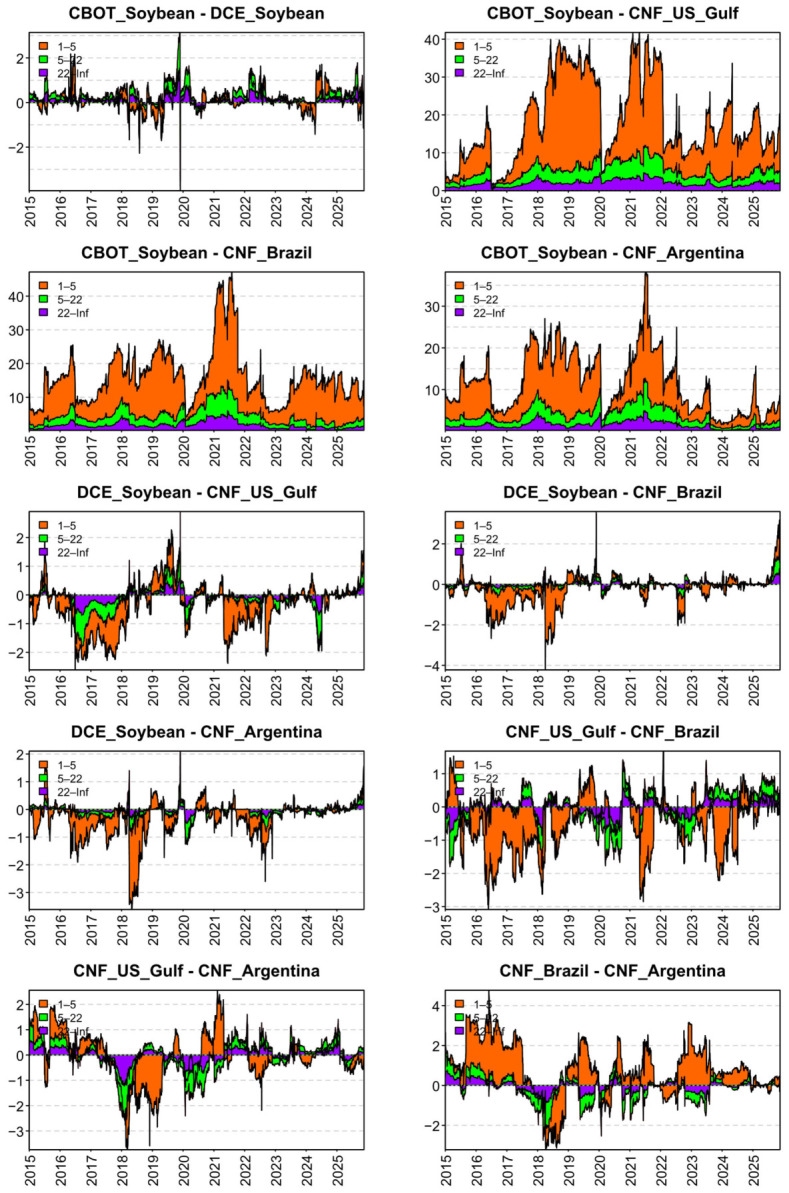
Pairwise network risk spillovers across frequency bands (absolute returns). Note: CBOT = Chicago Board of Trade soybean futures; DCE = Dalian Commodity Exchange soybean futures; CNF = Cost and Freight prices; positive (negative) values indicate net spillovers transmitted to (received from) other markets.

**Figure A5:**
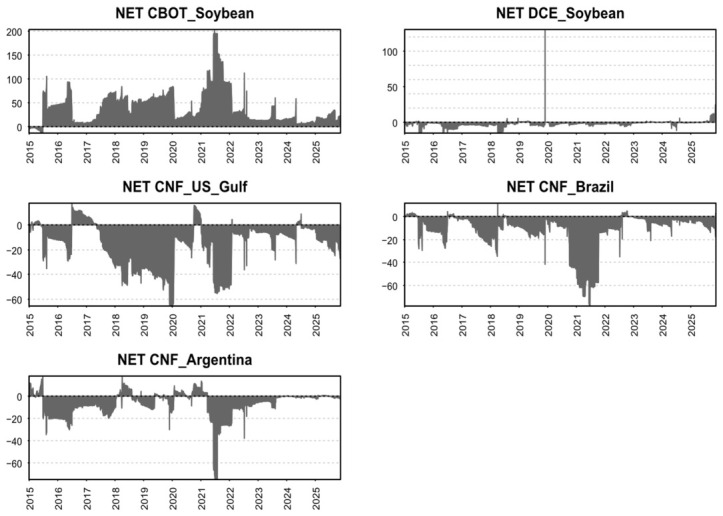
Dynamic net risk spillovers of each price series (squared returns). Note: CBOT = Chicago Board of Trade soybean futures; DCE = Dalian Commodity Exchange soybean futures; CNF = Cost and Freight prices.

**Figure A6:**
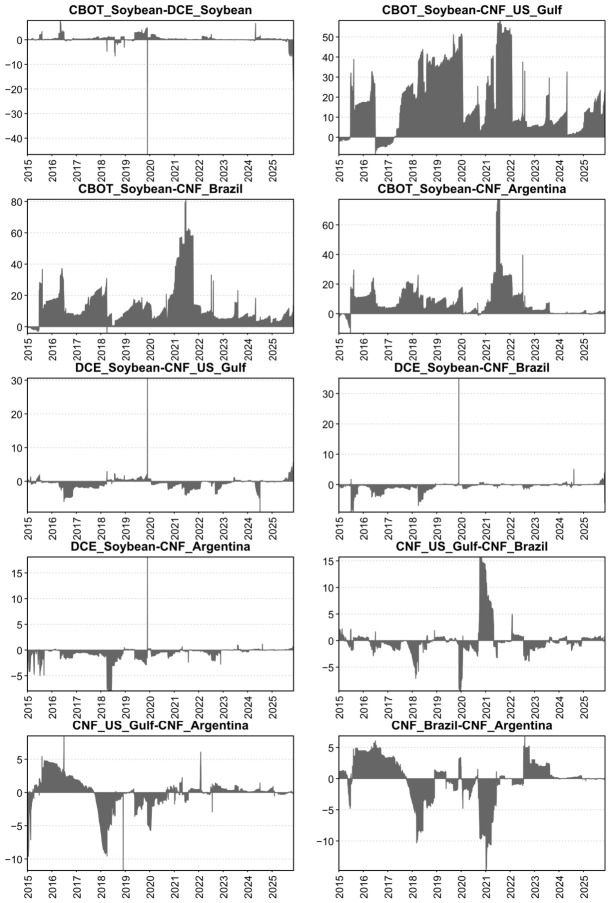
Pairwise risk spillovers among soybean prices (squared returns). Note: CBOT = Chicago Board of Trade soybean futures; DCE = Dalian Commodity Exchange soybean futures; CNF = Cost and Freight prices; positive (negative) values indicate net spillovers transmitted to (received from) other markets.

**Figure A7:**
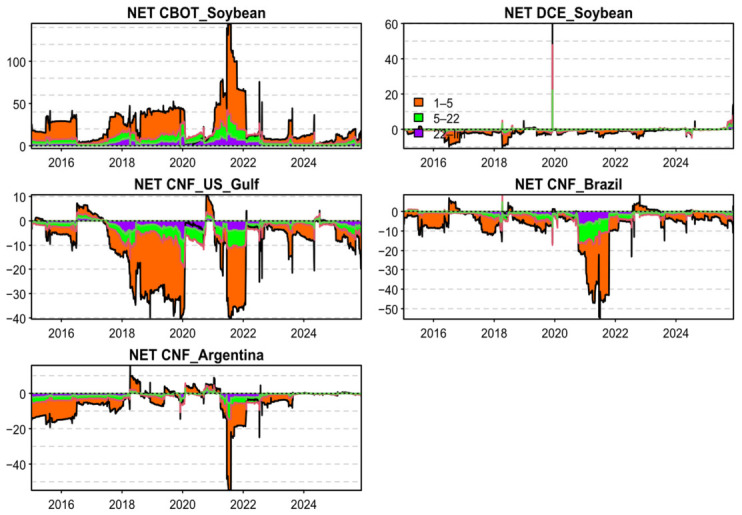
Pairwise network risk spillovers across frequency bands (squared returns). Note: CBOT = Chicago Board of Trade soybean futures; DCE = Dalian Commodity Exchange soybean futures; CNF = Cost and Freight prices.

**Figure A8:**
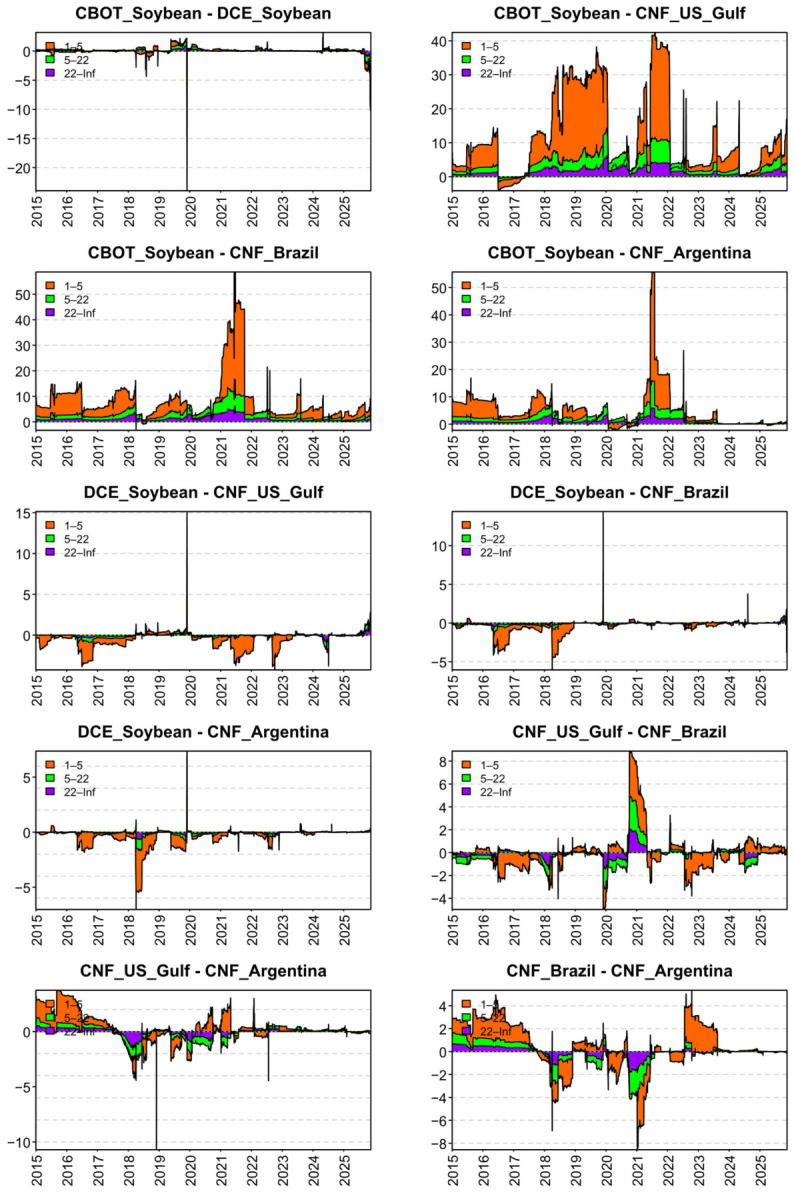
Pairwise network risk spillovers across frequency bands (squared returns). Note: CBOT = Chicago Board of Trade soybean futures; DCE = Dalian Commodity Exchange soybean futures; CNF = Cost and Freight prices; positive (negative) values indicate net spillovers transmitted to (received from) other markets.

**Figure A9:**
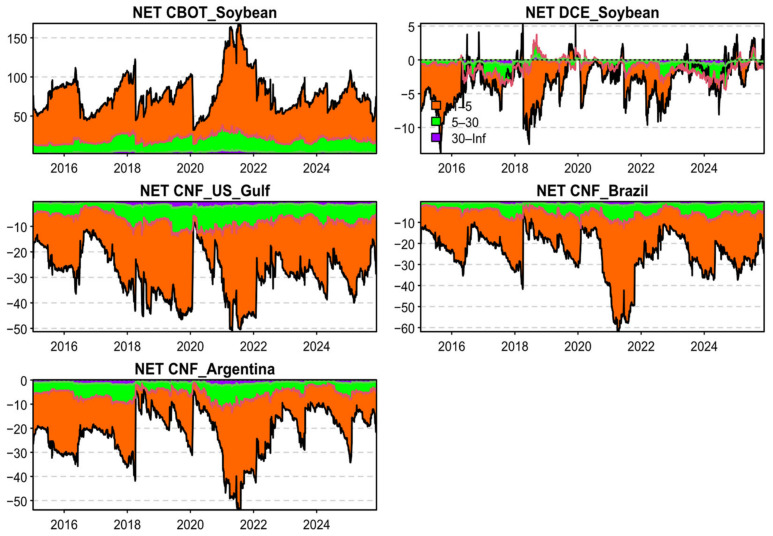
Pairwise network risk spillovers across frequency bands (relaxing long-term boundaries). Note: CBOT = Chicago Board of Trade soybean futures; DCE = Dalian Commodity Exchange soybean futures; CNF = Cost and Freight prices.

**Figure A10:**
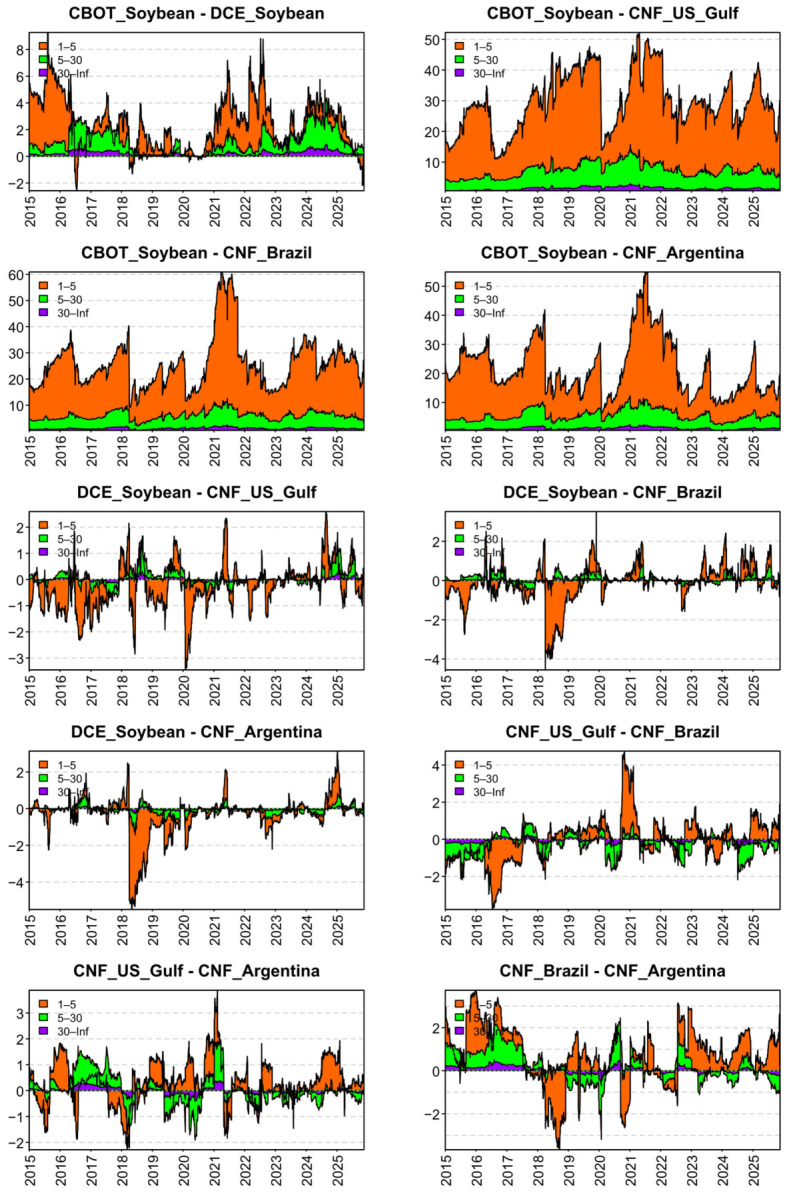
Pairwise network risk spillovers across frequency bands (relaxing long-term boundaries). Note: CBOT = Chicago Board of Trade soybean futures; DCE = Dalian Commodity Exchange soybean futures; CNF = Cost and Freight prices; positive (negative) values indicate net spillovers transmitted to (received from) other markets.

**Figure A11:**
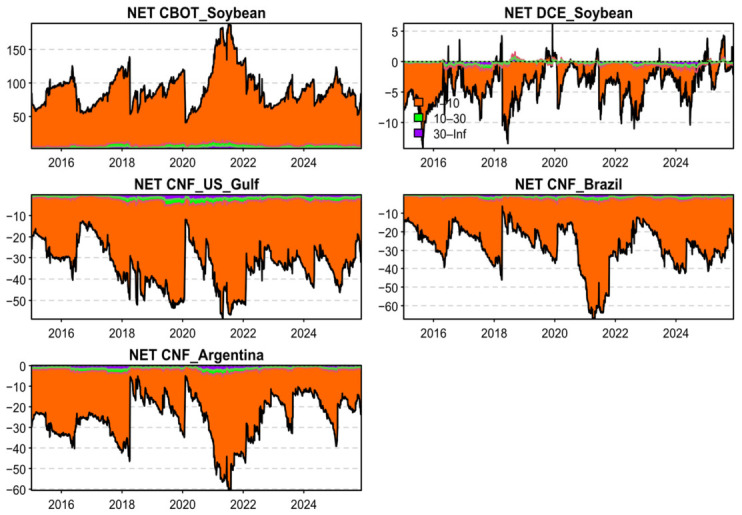
Pairwise network risk spillovers across frequency bands (relaxing medium- and long-term boundaries). Note: CBOT = Chicago Board of Trade soybean futures; DCE = Dalian Commodity Exchange soybean futures; CNF = Cost and Freight prices.

**Figure A12:**
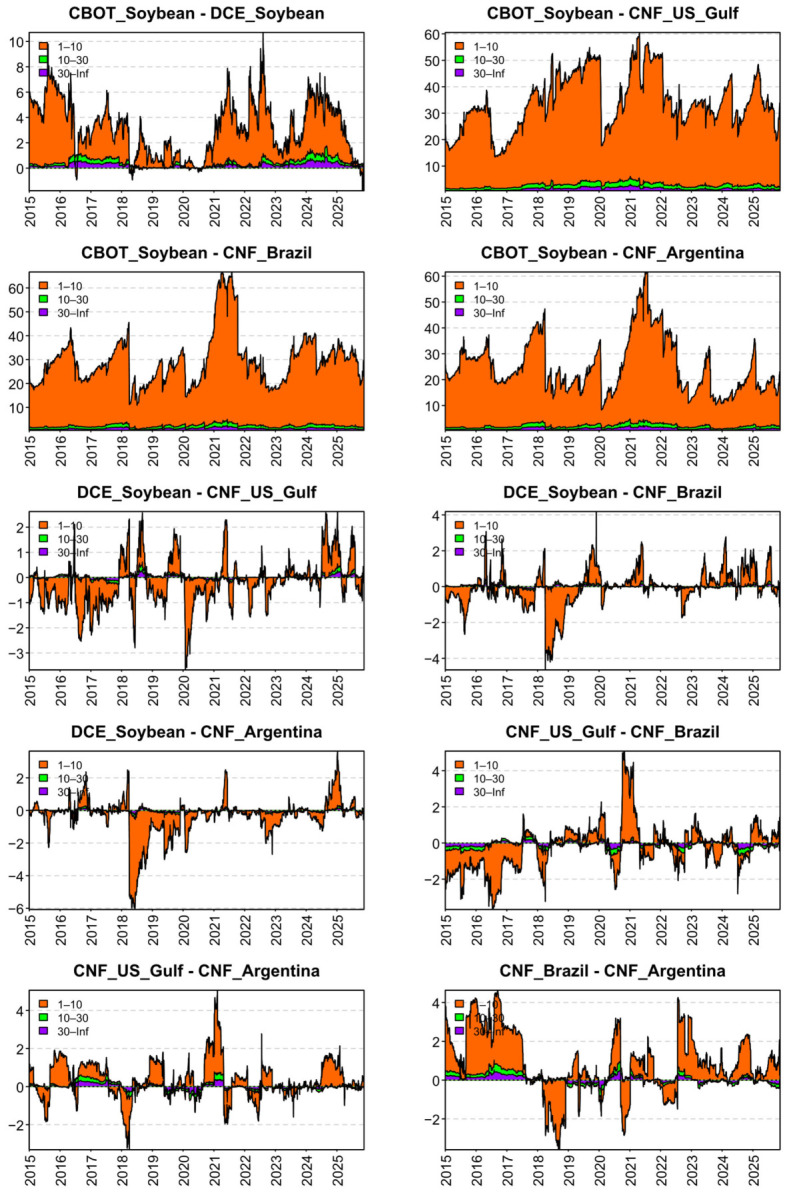
Pairwise network risk spillovers across frequency bands (relaxing medium- and long-term boundaries). Note: CBOT = Chicago Board of Trade soybean futures; DCE = Dalian Commodity Exchange soybean futures; CNF = Cost and Freight prices; positive (negative) values indicate net spillovers transmitted to (received from) other markets.

**Figure A13:**
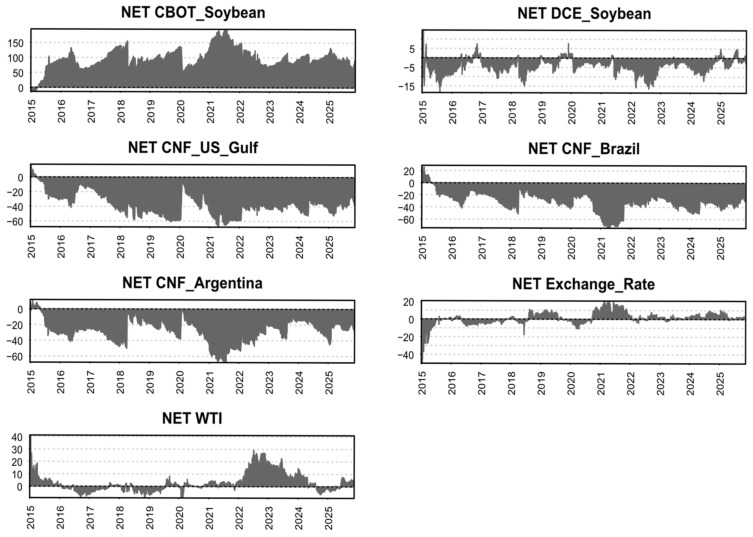
Dynamic net risk spillovers of each price series (with additional common shocks). Note: CBOT = Chicago Board of Trade soybean futures; DCE = Dalian Commodity Exchange soybean futures; CNF = Cost and Freight prices; WTI = West Texas Intermediate.

**Figure A14:**
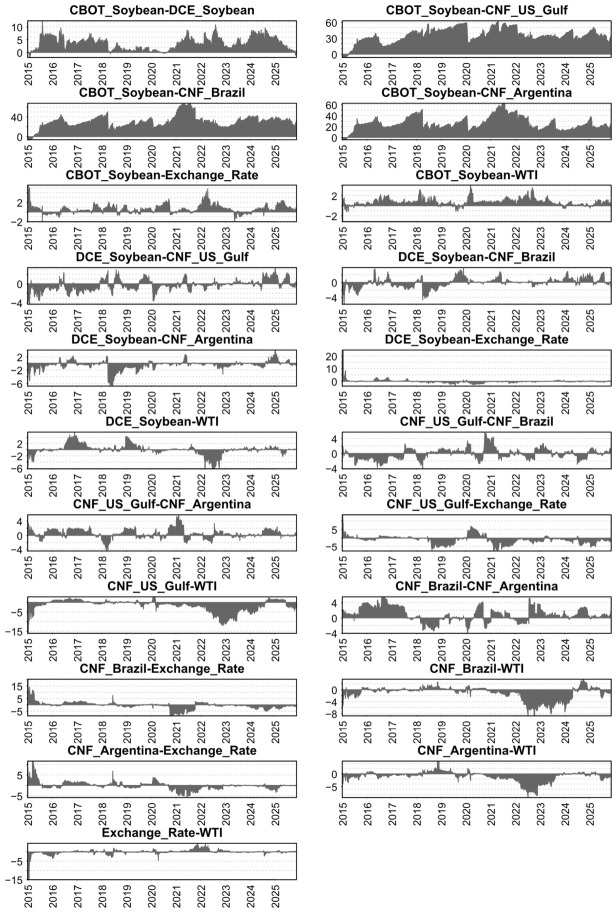
Pairwise risk spillovers among soybean prices (with additional common shocks). Note: CBOT = Chicago Board of Trade soybean futures; DCE = Dalian Commodity Exchange soybean futures; CNF = Cost and Freight prices; WTI = West Texas Intermediate; positive (negative) values indicate net spillovers transmitted to (received from) other markets.

**Figure A15:**
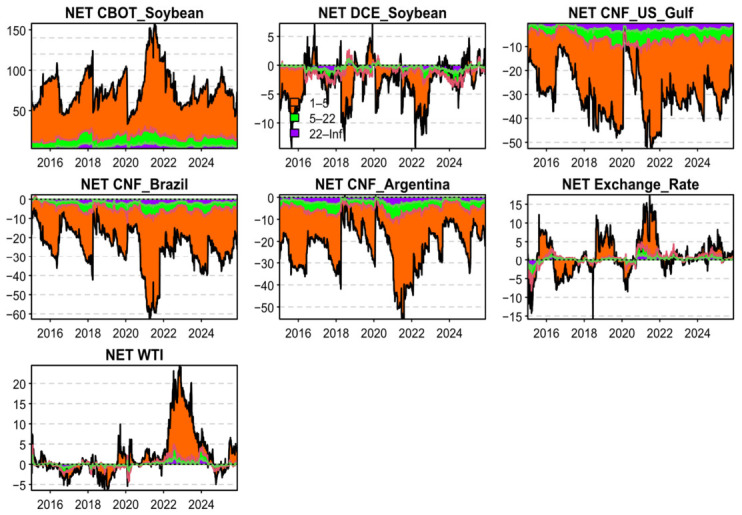
Pairwise network risk spillovers across frequency bands (with additional common shocks). Note: CBOT = Chicago Board of Trade soybean futures; DCE = Dalian Commodity Exchange soybean futures; CNF = Cost and Freight prices; WTI = West Texas Intermediate.

**Figure A16:**
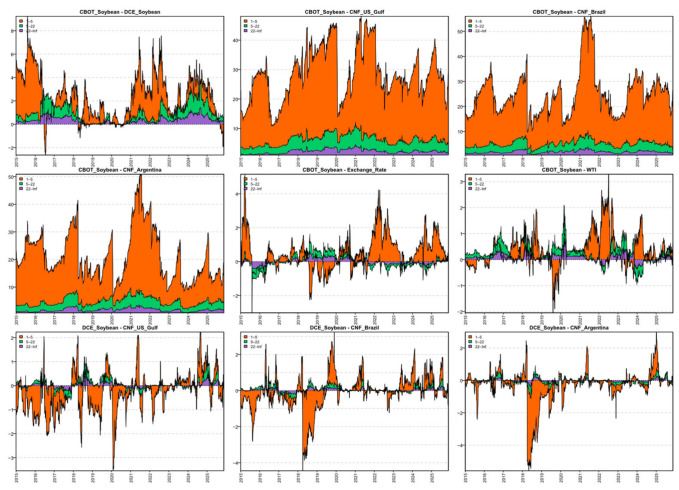

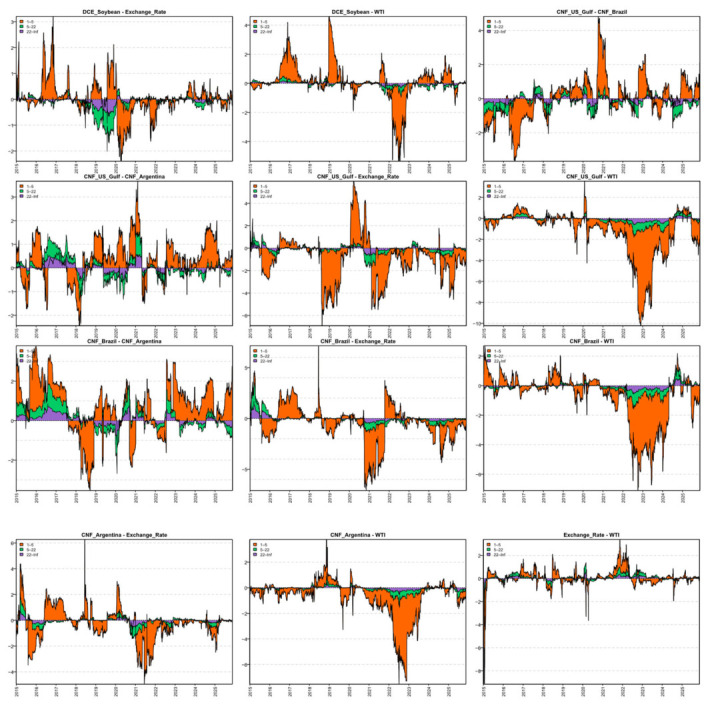
Pairwise network risk spillovers across frequency bands (with additional common shocks). Note: CBOT = Chicago Board of Trade soybean futures; DCE = Dalian Commodity Exchange soybean futures; CNF = Cost and Freight prices; WTI = West Texas Intermediate; positive (negative) values indicate net spillovers transmitted to (received from) other markets.

**Figure A17:**
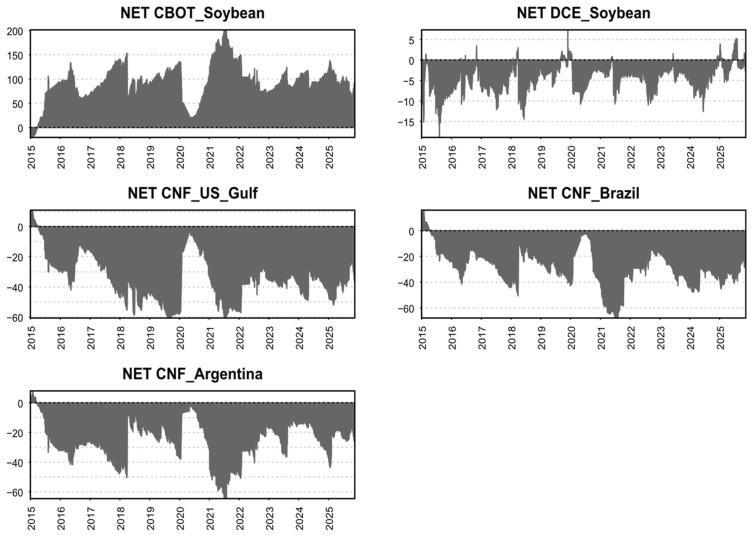
Dynamic net risk spillovers of each price series (excluding extreme event windows). Note: CBOT = Chicago Board of Trade soybean futures; DCE = Dalian Commodity Exchange soybean futures; CNF = Cost and Freight prices.

**Figure A18:**
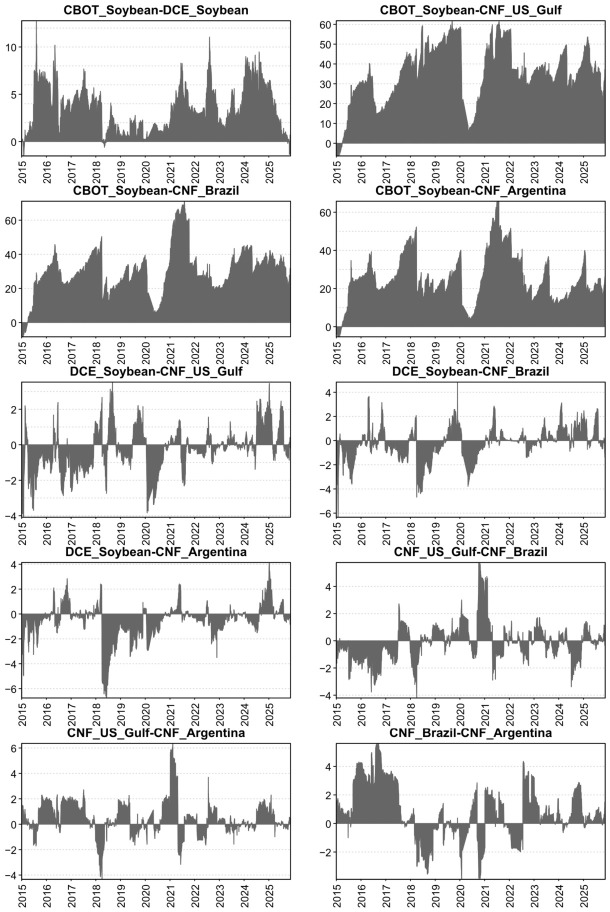
Pairwise risk spillovers among soybean prices (excluding extreme event windows). Note: CBOT = Chicago Board of Trade soybean futures; DCE = Dalian Commodity Exchange soybean futures; CNF = Cost and Freight prices; positive (negative) values indicate net spillovers transmitted to (received from) other markets.

**Figure A19:**
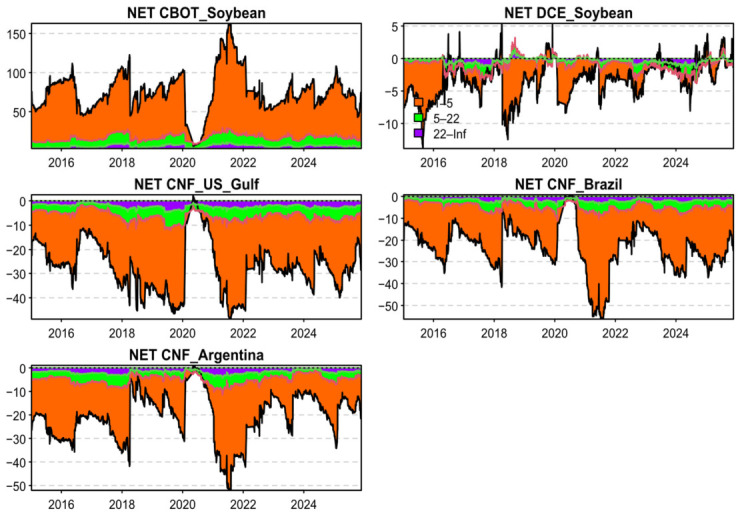
Pairwise network risk spillovers across frequency bands (excluding extreme event windows). Note: CBOT = Chicago Board of Trade soybean futures; DCE = Dalian Commodity Exchange soybean futures; CNF = Cost and Freight prices.

**Figure A20:**
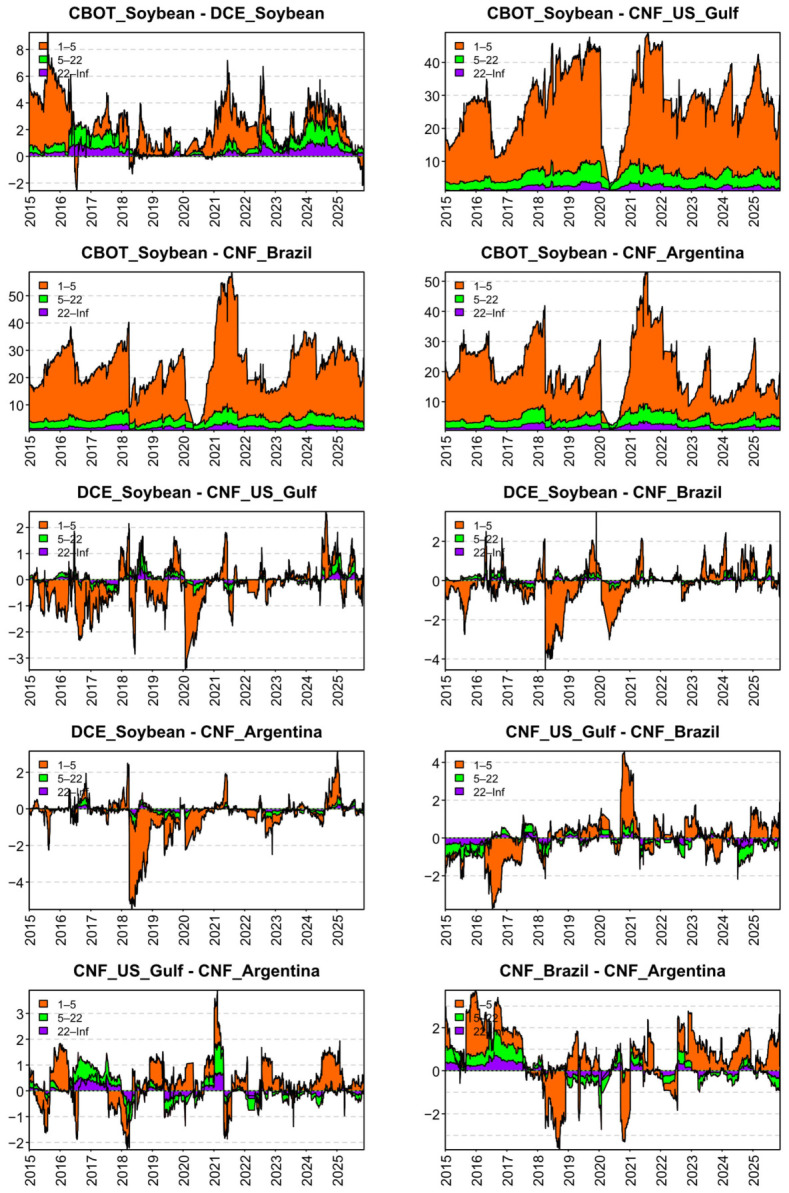
Pairwise network risk spillovers across frequency bands (excluding extreme event windows). Note: CBOT = Chicago Board of Trade soybean futures; DCE = Dalian Commodity Exchange soybean futures; CNF = Cost and Freight prices; positive (negative) values indicate net spillovers transmitted to (received from) other markets.

## Data Availability

The datasets generated and/or analyzed during the current study are available from the corresponding author on reasonable request.

## Abbreviations

The following abbreviations are used in this manuscript:

TVP-VAR-DY model	Time-varying parameter vector autoregression with the spillover connectedness measures of Diebold and Yılmaz
TVP-VAR-BK model	Time-varying parameter vector autoregression with frequency-specific dynamic connectedness measures of Baruník and Křehlík
CBOT	Chicago Board of Trade soybean futures;
DCE	Dalian Commodity Exchange soybean futures
CNF	Cost and Freight prices
TSI	Total Spillover Index

## Appendix A

**Table A1:** Model specification and parameter settings.

Item	Specification
Variables	*CBOT_Soybean*, *DCE_Soybean*, *CNF_US_Gulf*, *CNF_Brazil*, *CNF_Argentina*
Sample period	6 January 2015–24 November 2025
Frequency	Daily
Number of observations	2426
Data source	Wind database
VAR lag order	1
FEVD horizon	20
Estimation framework	TVP-VAR
Estimation method	Kalman filter
Connectedness measure (time domain)	TVP-VAR-DY
Connectedness measure (frequency domain)	TVP-VAR-BK
Frequency bands	1–5 days; 5–22 days; >22 days
Time-varying parameter setting	kappa1 = 0.99; kappa2 = 0.99
Prior	BayesPrior

## References

[1] Amiti M., Redding S.J., Weinstein D.E. (2019). The impact of the 2018 tariffs on prices and welfare. J. Econ. Perspect..

[2] Fajgelbaum P.D., Goldberg P.K., Kennedy P.J., Khandelwal A.K. (2020). The return to protectionism. Q. J. Econ..

[3] Caldara D., Iacoviello M., Molligo P., Prestipino A., Raffo A. (2020). The economic effects of trade policy uncertainty. J. Monet. Econ..

[4] Carter C.A., Steinbach S. (2020). The impact of retaliatory tariffs on agricultural and food trade. Appl. Econ. Perspect. Policy.

[5] Grant J.H., Arita S., Beckman J., Kuhn B., Sands A. (2021). Agricultural exports and retaliatory trade actions. Appl. Econ. Perspect. Policy.

[6] Sabala E., Devadoss S. (2019). Impacts of Chinese tariff on world soybean markets. J. Agric. Resour. Econ..

[7] Adjemian M.K., Smith A., He W. (2021). Estimating the market effect of a trade war: The case of soybean tariffs. Food Policy.

[8] Bandyopadhyay A., Rajib P. (2023). The impact of Sino–US trade war on price discovery of soybean: A double-edged sword. J. Futures Mark..

[9] Wen T., Li P., Chen L., An Y. (2023). Market reactions to trade friction between China and the United States: Evidence from the soybean futures market. J. Manag. Sci. Eng..

[10] Wang M., Liu D., Wang Z., Li Y. (2023). Structural evolution of global soybean trade network and the Implications to China. Foods.

[11] Diebold F.X., Yilmaz K. (2009). Measuring financial asset return and volatility spillovers, with Application to Global Equity Markets. Econ. J..

[12] Diebold F.X., Yilmaz K. (2012). Better to give than to receive: Predictive directional measurement of volatility spillovers. Int. J. Forecast..

[13] Diebold F.X., Yılmaz K. (2014). On the network topology of variance decompositions: Measuring the connectedness of financial firms. J. Econom..

[14] Antonakakis N., Chatziantoniou I., Gabauer D. (2020). Refined measures of dynamic connectedness based on time-varying parameter vector autoregressions. J. Risk Financ. Manag..

[15] Baruník J., Křehlík T. (2018). Measuring the frequency dynamics of financial connectedness and systemic risk. J. Financ. Econom..

[16] Baruník J., Ellington M. (2024). Persistence in financial connectedness and systemic risk. Eur. J. Oper. Res..

[17] U.S. Department of Agriculture (2025). Oilseeds: World Markets and Trade.

[18] Denicoff M.R., Prater M.E., Bahizi P. (2014). Soybean Transportation Profile.

[19] Cao Y., Cheng S. (2021). Impact of COVID-19 outbreak on multi-scale asymmetric spillovers between food and oil prices. Resour. Policy.

[20] Xue H., Du Y., Gao Y., Su W. (2024). Spatial price transmission and volatility spillovers. Foods.

[21] Barboza Martignone G.M., Ghosh B., Behrendt K., Paparas D. (2024). Leadership shift in the global soybean market: Dynamic connectedness approach (TVP-VAR). Heliyon.

[22] Guo J., Wu D.D., Zhang K., Luo C. (2022). Linkage relationship between China and US futures market during trade friction: The case of soybean, soybean oil and soybean meal. Appl. Econ. Lett..

[23] Lu X., Huang N., Mo J. (2024). Time-varying causalities from the COVID-19 media coverage to the dynamic spillovers among the cryptocurrency, the clean energy, and the crude oil. Energy Econ..

[24] Nasir M.A., Nugroho A.D., Lakner Z. (2022). Impact of the Russian–Ukrainian conflict on global food crops. Foods.

[25] Ouyang R., Chen X., Fang Y., Zhao Y. (2022). Systemic risk of commodity markets: A dynamic factor copula approach. Int. Rev. Financ. Anal..

[26] Cui J., Maghyereh A. (2023). Higher-order moment risk connectedness and optimal investment strategies between international oil and commodity futures markets: Insights from the COVID-19 pandemic and Russia-Ukraine conflict. Int. Rev. Financ. Anal..

[27] Chen J., Liang Z., Ding Q., Ren X., Wu A. (2023). Dynamic connectedness across energy and metal futures markets during the COVID-19 pandemic: New evidence from a time-varying spillover index. Resour. Policy.

[28] Lawson J., Alam R., Etienne X.L. (2021). Speculation and food-grain prices. Appl. Econ..

[29] Goswami A., Karali B. (2022). The impact of fundamentals on volatility measures of agricultural substitutes. J. Agric. Appl. Econ..

[30] Avileis F., Mallory M.L. (2021). The impact of Brazil on global grain dynamics: A study on cross-market volatility spillovers. Agric. Econ..

[31] Lv W.H., Ye L., Wang L. (2022). Changes of China’s soybean import market power and influencing factors. Appl. Econ. Lett..

[32] El-Shafei A.M.A., Altaha’at E.S.Y., Metawea A.A., Fouad E.M., Ayoub A., Elesawy A.E., Eliw M. (2024). An effective pragmatic analysis of soybean imports demand in Egypt: Impact of price and expenditure. J. Anim. Plant Sci..

[33] Zhang W., Zhan Y., Liang Y. (2025). Financial openness, major events, and exchange rate linkage: Empirical analysis based on the DCC-MIDAS model. Int. Rev. Financ. Anal..

